# The association between endothelial activation and stress Index and the development and prognosis of acute kidney injury in elderly patients with critical illness

**DOI:** 10.1080/0886022X.2025.2577174

**Published:** 2025-11-04

**Authors:** Zhiyuan Zhang, Yubo Li, Yang Wei, Yu Yang, Zixin Luo, Jiahui Xie, Qinglin Xu, Kang Zou, Jie Wang

**Affiliations:** a The First Clinical Medical College, Gannan Medical University, Ganzhou City, Jiangxi Province, the People’s Republic of China; b Department of Critical Care Medicine, the First Affiliated Hospital of Gannan Medical University, Ganzhou City, Jiangxi Province, the People’s Republic of China; c Department of Hepatobiliary Surgery, Affiliated Hospital of Qinghai University, Xining City, Qinghai Province, the People’s Republic of China; d Department of Critical Care Medicine, The Second Hospital of Xingguo County, Ganzhou City, Jiangxi Province, the People’s Republic of China

**Keywords:** Acute kidney injury, association, elderly, endothelial activation and stress index, MIMIC-IV

## Abstract

This study investigated the link between the endothelial activation and stress index (EASIX) and acute kidney injury (AKI) development and prognosis in elderly critically ill patients. Using the MIMIC-IV database, we conducted a retrospective cohort study including 12,122 ICU patients aged ≥65 years, of whom 9,124 developed AKI. Patients were divided into three groups based on EASIX scores. We compared the baseline characteristics, mortality rates, and clinical outcomes across groups. Multivariable Cox regression analysis assessed the association between EASIX and AKI development and short-term outcomes, adjusting for confounders. Kaplan–Meier curves and subgroup analyses were performed. Dose-response modeling, threshold effect analysis, and E-value analysis were also conducted. Results showed that patients with the highest EASIX scores had significantly higher mortality rates, with HRs for 28-day mortality of 1.69 (95% CI: 1.47–1.95, *p* < 0.001), in-ICU mortality of 1.55 (95% CI: 1.34–1.79, *p* < 0.001), and in-hospital mortality of 1.42 (95% CI: 1.13–1.79, *p* = 0.003). Kaplan–Meier curves indicated lower survival probabilities with higher EASIX values (log-rank *p* < 0.001). Dose-response analysis revealed a nonlinear relationship with a threshold effect at an EASIX value of around 175. Subgroup analysis found a significant interaction in the CHF subgroup (*p* < 0.001), suggesting the increased vulnerability to elevated EASIX. In conclusion, elevated EASIX is significantly associated with AKI development and adverse short-term outcomes in elderly critically ill patients, indicating its potential as an index for identifying high-risk patients.

## Introduction

Acute kidney injury (AKI) is a critical complication frequently encountered in intensive care units (ICUs), characterized by its high incidence and mortality rates, particularly among the elderly demographic. The incidence of AKI in critically ill patients can range from 20% to 50%, imposing a considerable burden on healthcare resources and significantly extending patients’ hospital stays, thereby escalating the medical costs [1,2]. The effective identification of high-risk patients and the enhancement of their prognoses have emerged as pivotal issues in clinical practice [3,4].

The elderly population is particularly vulnerable to AKI due to age-related physiological changes, including a progressive decline in renal function and reserve, which diminishes their ability to withstand acute insults [5,6]. Comorbidities such as diabetes, hypertension, and cardiovascular diseases, which are prevalent in older adults, further complicate the management and prognosis of AKI [7,8]. Studies indicate that AKI in elderly patients is associated with the higher mortality rates and poorer outcomes compared to younger patients [9,10]. This multifactorial nature of AKI necessitates a sophisticated approach to diagnosis, risk stratification, and treatment [11,12].

The pathophysiology of AKI is complex, involving alterations in renal hemodynamics and inflammatory responses. Dysregulation of factors affecting renal perfusion can lead to impaired glomerular filtration and overall renal blood flow [12,13]. Inflammatory cells infiltrate renal tissue, releasing cytokines such as tumor necrosis factor-alpha (TNF-α) and interleukin-6 (IL-6), which activate intracellular signaling pathways like nuclear factor kappa-light-chain-enhancer of activated B cells (NF-κB) [3,4]. This cascade can result in endothelial dysfunction, apoptosis of tubular cells, and renal hypertension [14]. Given the intricate nature of AKI, there is an urgent need for reliable biomarkers to facilitate early diagnosis and risk stratification [15,16]. Traditional biomarkers, such as serum creatinine and urine output, often fail to provide timely and precise information, underscoring the necessity for novel markers [17,18].

In recent years, the Endothelial Activation and Stress Index (EASIX) has garnered attention as a potential biomarker for AKI. EASIX is calculated from easily accessible clinical parameters, including lactate dehydrogenase, platelet count, and serum creatinine, making it straightforward to evaluate. This index was first proposed in 2017. It is used to predict mortality in patients with acute graft-versus-host disease (GVHD) after allogeneic hematopoietic stem cell transplantation [19]. Although its prognostic value has been established in various diseases—such as atrial fibrillation and acute pancreatitis—research on the application of EASIX in the context of AKI, particularly among elderly patients, it remains limited [20,21]. Given the high incidence and poor prognosis of AKI in this demographic, investigating the role of EASIX could provide valuable insights into its potential use as a prognostic biomarker. The research aims to explore the association between the EASIX score and short-term prognosis in elderly patients with AKI. It is hypothesized that the higher EASIX values are linked to increased mortality rates and adverse clinical outcomes. Elucidating this association could enhance clinical decision-making, reduce patient mortality, and improve the overall quality of healthcare for this vulnerable population.

## Methods

### Data sources and study setting

This study employs a retrospective cohort design and utilizes data from the Medical Information Mart for Intensive Care IV (MIMIC-IV) database. This comprehensive and publicly accessible database contains detailed electronic health records (EHRs) of patients admitted to the ICUs at Beth Israel Deaconess Medical Center in Boston, MA, USA, spanning from 2008 to 2019. The study’s design and reporting adhere to the Strengthening the Reporting of Observational Studies in Epidemiology (STROBE) guidelines, ensuring methodological rigor and transparency. Given the retrospective nature of the study and the de-identified status of the data, informed consent was waived, and the study was exempt from institutional review board approval. Access to the database requires researchers to complete the CITI program and successfully pass the ‘Conflict of Interest’ and ‘Data or Sample Research Only’ exams (ID: 3990616).

### Inclusion and exclusion criteria for the study population

**Figure 1.**
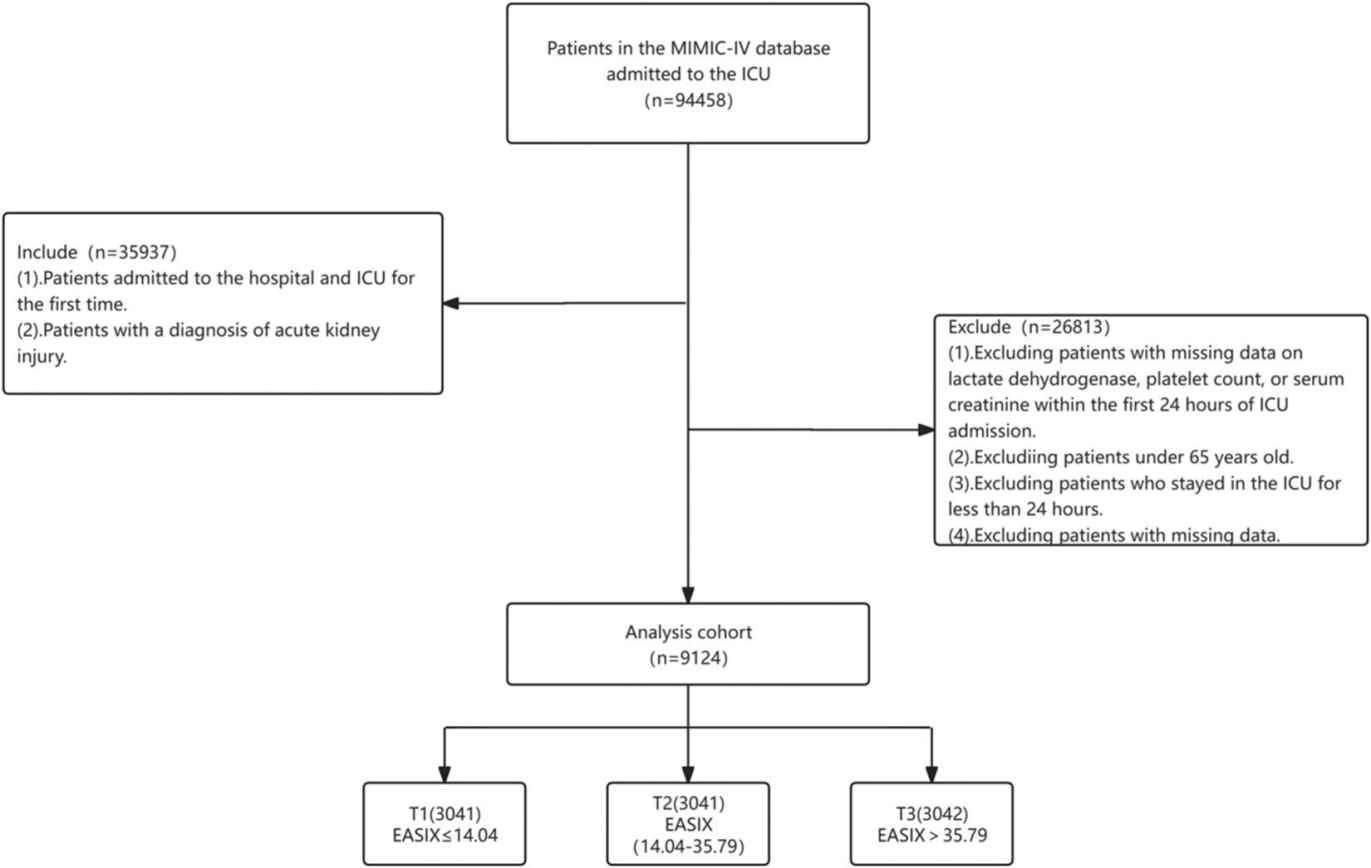
Flowchart of study patients.

The study population was extracted from the MIMIC-IV database and consisted of adult patients aged 65 years and older who were admitted to the ICU with a diagnosis of AKI. The inclusion criteria were as follows: (1) patients with their first hospital admission and first ICU admission and (2) a diagnosis of AKI established according to the kidney disease: Improving Global Outcomes (KDIGO) criteria. The exclusion criteria included: (1) lack of data for lactate dehydrogenase (LDH), platelet count, or serum creatinine levels within the first 24 h of ICU admission, (2) age under 65 years, (3) ICU stays lasting less than 24 h, and (4) incomplete data records (Figure 1). Ultimately, the final cohort consisted of 9,124 patients with complete data available for analysis.

### Covariates

The analysis incorporated the following covariates: age, sex, ethnicity, weight, WBC, RBC, hemoglobin levels, sodium, potassium, total calcium, chloride, glucose, total bilirubin, BUN, SOFA, CCI, APSIII, and OASIS. Additionally, the study considered the use of ventilation, CRRT, vasopressin, and diuretics, as well as the presence of hypertension, diabetes, liver disease, myocardial infarction, congestive heart failure, cerebrovascular disease, chronic pulmonary disease, malignant cancer, and sepsis.

### EASIX definitions

The Endothelial Activation and Stress Index (EASIX) score (lactic dehydrogenase [LDH; U/L] × creatinine [mg/dL]/platelets [PLTs; 10^9^ cells/L]) [19].

### Research endpoint event

The principal endpoint of the research was the all-cause mortality rate at 28 days. Secondary endpoints encompassed the mortality rates within the ICU and overall, in-hospital mortality, 90-day mortality mortality, and 365-day mortality.

### Statistical analysis

A statistical analysis was performed to investigate the association between the EASIX and short-term outcomes in elderly patients experiencing AKI. Baseline characteristics were summarized using descriptive statistics, with continuous variables expressed as mean ± standard deviation (SD) or median (interquartile range, IQR), and categorical variables reported as frequencies and percentages. The EASIX values were multiplied by 10 before analysis to enhance data readability. To evaluate the association between EASIX and mortality outcomes—specifically 28-day mortality, in-ICU mortality, and in-hospital mortality—multivariable Cox regression models were utilized. The variables for the multivariate Cox regression analysis were chosen based on the existing literature and clinical experience. In Model 1, demographic factors like age, gender, and race were included as they are common variables that can influence patient prognosis. These factors help control for potential confounding effects on the relationship between AKI occurrence and mortality. Model 2 builds on Model 1 by adding clinical variables related to AKI, such as comorbidities (e.g., cardiovascular disease, diabetes), vital signs at ICU admission, and key lab indicators like hemoglobin. The selection of these variables was informed by clinical practice. Kaplan–Meier survival curves were generated to depict variations in survival probabilities across EASIX tertiles, with log-rank tests employed to evaluate the statistical significance. Subgroup analyses were conducted to investigate the potential interactions between EASIX and mortality outcomes within predefined subgroups stratified by sex, SOFA, CCI, hypertension, diabetes, congestive heart failure, and potassium levels. A two-sided *p*-value of less than 0.05 was deemed statistically significant. Analyses were performed using R version 4.4.2 (R Foundation) and Free Statistics Software version 2.1 and a *p*-value below 0.05 indicating statistical significance.

## Results

### Population and baseline characteristics

A total of 9,124 elderly patients with AKI were enrolled and stratified into three EASIX-based tertiles: T1 (lowest:EASIX ≤ 14.04), T2 (intermediate:14.04 < EASIX ≤ 35.79), and T3 (highest:EASIX > 35.79). Table 1 outlines the baseline characteristics of the elderly AKI population: Average age was 77.2 years, with 44.4% female, 55.6% male, and 66.5% White patients. Mortality rates were 21.7% in 28 days, 8.0% in-hospital, and 22.4% in the ICU. Mean hospital stay was 15.5 days, and ICU stay was 6.6 days. Participants in the highest-EASIX tertile (T3) had the longest stays and highest mortality rate. T3 patients displayed the elevated heart and respiratory rates; higher SOFA, CCI, APSIII, and OASIS scores; increased WBC, potassium, glucose, bilirubin, and BUN levels; and lower MBP, RBC, hemoglobin, sodium, calcium, and chloride levels. They also exhibited greater burdens of hypertension, diabetes, and sepsis, and were more likely to receive CRRT and vasopressors. Supplementary Table 1 enrolled 12,122 elderly patients (mean age 77.1 years; 45.2% female, 54.8% male; 66.9% White). Across EASIX tertiles, the T3 group had the heaviest disease burden: highest critical-care scores, inflammatory and metabolic markers, more comorbidities, greater use of CRRT and vasopressors, the longest hospital and ICU stay, and an AKI incidence of 85.4%.

**Table 1.** Baseline characteristics of participants and outcome parameters.

	Total (*n* = 9,124)	Endothelial activation and stress index			***p*** value
Variables		T1	T2	T3	
		(*n* = 3,041)	(*n* = 3,041)	(*n* = 3,042)	
**Age (years)**	77.2 ± 8.0	77.3 ± 8.1	77.6 ± 7.9	76.7 ± 7.9	**< 0.001**
**Sex** (%)					**< 0.001**
F	4,053 (44.4)	1,685 (55.4)	1,197 (39.4)	1,171 (38.5)	
M	5,071 (55.6)	1,356 (44.6)	1,844 (60.6)	1,871 (61.5)	
**Ethnicity (%)**					**< 0.001**
OTHER	3,055 (33.5)	939 (30.9)	1,017 (33.4)	1,099 (36.1)	
WHITE	6,069 (66.5)	2,102 (69.1)	2,024 (66.6)	1,943 (63.9)	
**Weight (Kg)**	79.3 ± 21.4	75.0 ± 20.8	80.6 ± 21.2	82.2 ± 21.4	**< 0.001**
**Vital signs**					
Heart rate (bpm)	89.1 ± 21.0	89.2 ± 20.8	88.2 ± 20.9	89.8 ± 21.1	**0.010**
Respiration (bpm)	20.0 ± 6.5	19.9 ± 6.2	19.7 ± 6.5	20.5 ± 6.6	**< 0.001**
Spo2 (%)	96.6 ± 10.2	96.7 ± 3.9	96.6 ± 5.1	96.4 ± 16.5	0.681
MBP (mmHg)	80.7 ± 19.3	82.8 ± 19.4	80.7 ± 18.7	78.6 ± 19.6	**< 0.001**
**Scoring system, points**					
SOFA	6.2 ± 3.6	4.2 ± 2.8	5.9 ± 3.0	8.4 ± 3.5	**< 0.001**
CCI	6.9 ± 2.6	6.3 ± 2.5	6.8 ± 2.6	7.5 ± 2.6	**< 0.001**
Apsiii	53.8 ± 21.4	45.7 ± 17.8	51.5 ± 19.7	64.3 ± 22.3	**< 0.001**
Oasis	35.3 ± 8.5	34.5 ± 7.9	34.8 ± 8.3	36.7 ± 9.1	**< 0.001**
**Comorbidities**					
Hypertension (%)	3,820 (41.9)	1,689 (55.5)	1,271 (41.8)	860 (28.3)	**< 0.001**
Diabetes (%)	3,318 (36.4)	915 (30.1)	1,151 (37.8)	1,252 (41.2)	**< 0.001**
Liver disease (%)	5,920 (64.9)	2,092 (68.8)	1,995 (65.6)	1,833 (60.3)	**< 0.001**
Myocardial infarct (%)	1,271 (13.9)	231 (7.6)	418 (13.7)	622 (20.4)	**< 0.001**
Congestive heart failure (%)	3,875 (42.5)	1,000 (32.9)	1,376 (45.2)	1,499 (49.3)	**< 0.001**
Cerebrovascular disease (%)	2,034 (22.3)	724 (23.8)	688 (22.6)	622 (20.4)	**0.006**
Chronic pulmonary disease (%)	1,001 (11.0)	338 (11.1)	356 (11.7)	307 (10.1)	0.125
Malignant cancer (%)	1,961 (21.5)	690 (22.7)	653 (21.5)	618 (20.3)	0.079
Sepsis (%)	6,311 (69.2)	1,894 (62.3)	2,067 (68)	2,350 (77.3)	**< 0.001**
**Laboratory results**					
WBC (K/uL)	13.6 ± 11.9	13.1 ± 8.0	13.2 ± 10.7	14.3 ± 15.6	**< 0.001**
RBC (K/uL)	3.5 ± 0.8	3.6 ± 0.7	3.5 ± 0.8	3.3 ± 0.8	**< 0.001**
Hemoglobin (g/dL)	10.4 ± 2.2	10.7 ± 2.1	10.5 ± 2.3	10.0 ± 2.3	**< 0.001**
Sodium (mEq/L)	138.2 ± 5.7	138.0 ± 5.5	138.5 ± 5.3	138.2 ± 6.2	**< 0.001**
Potassium (mEq/L)	4.3 ± 0.8	4.1 ± 0.7	4.3 ± 0.7	4.5 ± 0.9	**< 0.001**
Calciumtotal (mg/dL)	8.3 ± 0.9	8.4 ± 0.8	8.3 ± 0.8	8.2 ± 0.9	**< 0.001**
Chloride (mEq/L)	103.7 ± 6.9	103.3 ± 6.6	104.5 ± 6.5	103.3 ± 7.6	**< 0.001**
Glucose (mg/dL)	154.1 ± 76.9	144.6 ± 60.5	156.2 ± 80.2	161.6 ± 86.7	**< 0.001**
Total bilirubin (mg/dL)	1.4 ± 2.9	1.0 ± 1.8	1.2 ± 2.5	1.9 ± 3.9	**< 0.001**
BUN (mg/dL)	34.1 ± 25.5	21.5 ± 12.6	30.7 ± 18.9	50.1 ± 31.7	**< 0.001**
**Interventions**					
Ventilation (%)	8,089 (88.7)	2,663 (87.6)	2,728 (89.7)	2,698 (88.7)	**0.032**
CRRT (%)	778 (8.5)	41 (1.3)	142 (4.7)	595 (19.6)	**< 0.001**
Vasopressin (%)	5,767 (63.2)	1,650 (54.3)	1,959 (64.4)	2,158 (70.9)	**< 0.001**
Diuretic (%)	2,296 (25.2)	700 (23)	898 (29.5)	698 (22.9)	**< 0.001**
**ACEI (%)**	1,820 (19.9)	689 (22.7)	664 (21.8)	467 (15.4)	**< 0.001**
**Statins (%)**	1,807 (19.8)	614 (20.2)	658 (21.6)	535 (17.6)	**< 0.001**
**Hospital stays**	15.5 ± 14.5	14.9 ± 13.4	15.2 ± 13.3	16.3 ± 16.5	**< 0.001**
**ICU stay**	6.6 ± 7.5	6.2 ± 7.2	6.6 ± 7.6	6.9 ± 7.8	**< 0.001**
**In-ICU mortality (%)**	2,048 (22.4)	439 (14.4)	573 (18.8)	1,036 (34.1)	**< 0.001**
**In-hosptial mortality (%)**	732 (8.0)	190 (6.2)	226 (7.4)	316 (10.4)	**< 0.001**
**28-day mortality (%)**	1,977 (21.7)	432 (14.2)	559 (18.4)	986 (32.4)	**< 0.001**

**Note:** Data are presented as the mean ± SD or median (IQR) for skewed variables, and numbers (proportions) for categorical variables. EASIX values were multiplied by 10 for analysis. *p* values less than 0.05 are expressed in bold.

bpm: beats per minute; MBP: Mean Blood Pressure; SOFA: Sequential Organ Failure Assessment score; CCI: Charlson Comorbidity Index; APSIII: Acute Physiology and Chronic Health Evaluation III score; OASIS: Outcome and Severity of Illness Score; WBC: White Blood Cell count; RBC: Red Blood Cell count; SCr: Serum Creatinine; BUN: Blood Urea Nitrogen. ACEI: angiotensin-converting enzyme inhibitors.

### Multivariable Cox regression analysis

Table 2 presents the hazard ratios (HR) and 95% confidence intervals (CI) for the primary outcome of 28-day mortality and the secondary outcomes of in-ICU and in-hospital mortality. For 28-day mortality, the unadjusted HRs were 1.24 (95% CI: 1.09–1.40, *p* = 0.001) for T2 and 2.12 (95% CI: 1.89–2.37, *p* < 0.001) for T3, using T1 as the reference. After adjusting for age, sex, and ethnicity (Model 1), the HRs were 1.25 (95% CI: 1.10–1.41, *p* = 0.001) and 2.22 (95% CI: 1.98–2.49, *p* < 0.001), respectively. After full adjustment (Model 2), the HRs were 1.22 (95% CI: 1.07–1.40, *p* = 0.003) and 1.69 (95% CI: 1.47–1.95, *p* < 0.001), respectively. Similar trends were observed for in-ICU and in-hospital mortality, with the highest risks in the T3 group, and *p*-values for trend < 0.05 across all models. Supplementary Table 2 (SOFA renal component excluded) shows that in Model 2 the T3 group had an HR of 1.73 (95% CI 1.51–1.99, *p* < 0.001) for 28-day mortality, indicating that high EASIX remains an independent risk factor even without the renal SOFA contribution. Similar upward trends were observed for in-ICU mortality (HR 1.56, 95% CI 1.36–1.79, *p* < 0.001) and in-hospital mortality (HR 1.56, 95% CI 1.25–1.94, *p* < 0.001). After full adjustment (Model 2), it is revealed that the T3 group had an OR of 1.65 (95% CI 1.42–1.90, *p* < 0.001) for AKI development (Supplementary Table 3), demonstrating that higher EASIX is associated with a greater likelihood of AKI.

**Table 2.** Multivariable Cox regression analysis of clinical outcomes.

	Unadjusted		Model 1		Model 2	
Variable	HR(95% CI)	*p* value	HR(95% CI)	*p* value	HR(95% CI)	*p* value
**Primary outcomes**						
**28-day mortality**						
T1	1(Ref)		1(Ref)		1(Ref)	
T2	1.24 (1.09 − 1.40)	**0.001**	1.25 (1.10 − 1.41)	**0.001**	1.22 (1.07 − 1.40)	**0.003**
T3	2.12 (1.89 − 2.37)	**<0.001**	2.22 (1.98 − 2.49)	**<0.001**	1.69 (1.47 − 1.95)	**<0.001**
P for trend		**<0.001**		**<0.001**		**<0.001**
**secondary oucomes**						
**In-ICU mortality**						
T1	1(Ref)		1(Ref)		1(Ref)	
T2	1.22 (1.08 − 1.38)	**0.002**	1.23 (1.09 − 1.40)	**0.001**	1.16 (1.02 − 1.32)	**0.029**
T3	2.03 (1.81 − 2.27)	**<0.001**	2.11 (1.88 − 2.36)	**<0.001**	1.55 (1.34 − 1.79)	**<0.001**
P for trend		**<0.001**		**<0.001**		**<0.001**
**In-hosptial mortality**						
T1	1(Ref)		1(Ref)		1(Ref)	
T2	1.16 (0.96 − 1.41)	0.123	1.17 (0.96 − 1.42)	0.121	1.23 (0.99 − 1.51)	0.051
T3	1.48 (1.23 − 1.77)	**<0.001**	1.58 (1.32 − 1.90)	**<0.001**	1.42 (1.13 − 1.79)	**0.003**
P for trend		**<0.001**		**<0.001**		**<0.001**
**90-day mortality**						
T1						
T2	1.05 (0.94 − 1.18)	0.400	1.03 (0.92 − 1.16)	0.627	1.04 (0.92 − 1.18)	0.523
T3	1.32 (1.19 − 1.47)	**<0.001**	1.37 (1.23 − 1.52)	**<0.001**	1.16 (1.02 − 1.32)	**0.022**
P for trend		**<0.001**		**<0.001**		**0.016**
**365-day mortality**						
T1						
T2	1.05 (0.94 − 1.18)	0.367	1.03 (0.92 − 1.15)	0.578	1.04 (0.93 − 1.17)	0.482
T3	1.32 (1.2 − 1.46)	**<0.001**	1.36 (1.23 − 1.51)	**<0.001**	1.16 (1.02 − 1.31)	**0.020**
P for trend		**<0.001**		**<0.001**		**0.015**

Note: Hazard ratios (HR) and 95% confidence intervals (CI) are presented for 28-day mortality, in-ICU mortality, in-hospital mortality, 90-day mortality, and 365-day mortality.

Unadjusted: Crude model without covariate adjustment.

**Model 1:** Adjusted for age, sex, and ethnicity.

**Model 2:** Adjusted for age, sex, ethnicity, weight, vital signs (heart rate, respiration rate, SpO_2_, mean blood pressure), scoring systems (SOFA, CCI, APSIII, OASIS), comorbidities (hypertension, diabetes, liver disease, myocardial infarct, congestive heart failure, cerebrovascular disease, chronic pulmonary disease, malignant cancer, sepsis), laboratory results (WBC, RBC, hemoglobin, sodium, potassium, calcium, chloride, glucose, total bilirubin, BUN), and interventions (ventilation, CRRT, vasopressin, diuretic, ACEI, statins).

*p* values less than 0.05 are expressed in bold.

### Kaplan–Meier survival analysis

**Figure 2.**
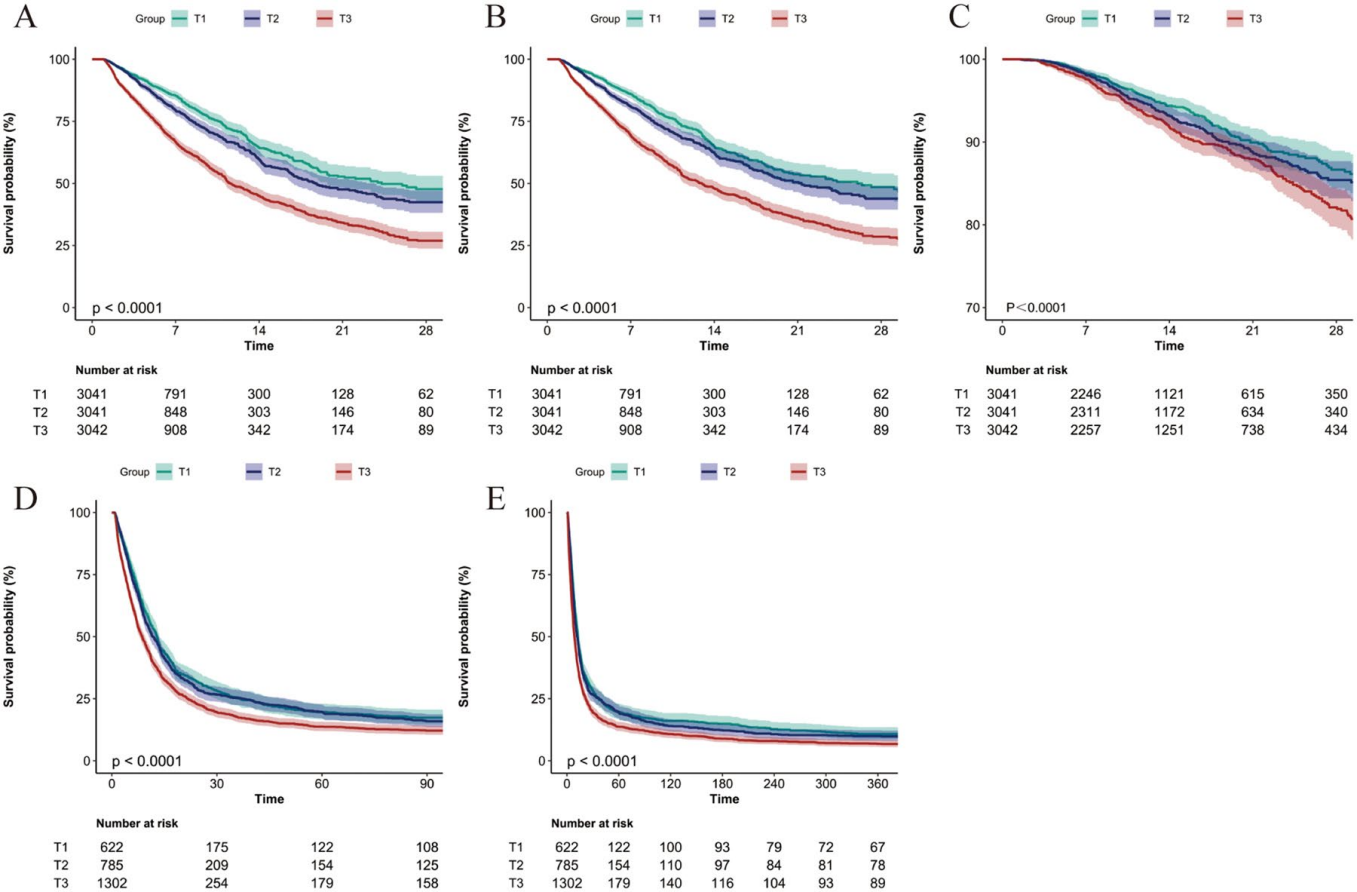
Kaplan–Meier survival curves for mortality outcomes in AKI patients: (A) 28-day mortality, (B) ICU mortality, (C) in-hospital mortality, (D) 90-day mortality, (E) 365-day mortality.

Utilizing the tertiles of the EASIX, Kaplan–Meier survival curves were constructed to assess 28-day mortality, in-ICU mortality, in-hospital mortality, 90-day mortality, and 365-day mortality, among patients with AKI (Figure 2(A)–(E)). The analysis indicated that individuals in the highest EASIX tertile exhibited the lowest survival rates at 28 days. Consistently, survival curves for both in-ICU mortality, in-hospital mortality, 90-day mortality, and 365-day mortality demonstrated that the elevated EASIX values correlated with reduced survival probabilities. These observed trends were statistically significant across all mortality outcomes, with *p*-values less than 0.05.

### Subgroup analysis

**Figure 3.**
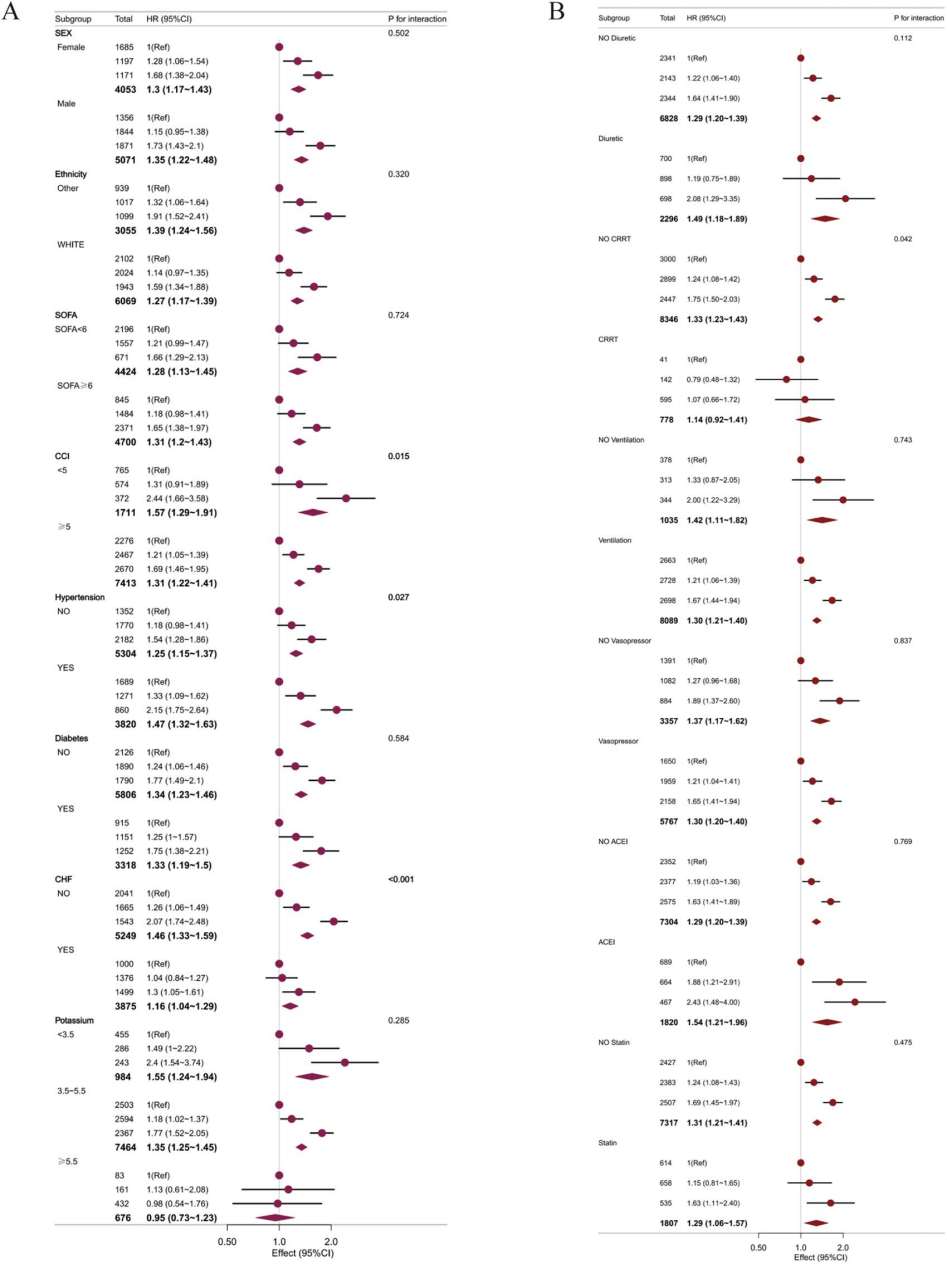
Subgroup analysis of EASIX and 28-day mortality: (A) laboratory parameters and comorbidities, and (B) treatment modalities.

Subgroup analyses were conducted by stratifying participants according to various variables, including sex, SOFA, CCI, hypertension, diabetes, CHF, and potassium levels. Following adjustment for the variables included in Model 2, a positive association between EASIX and 28-day mortality was observed across all subgroups (HR > 1 for each group). Notably, a significant interaction effect between EASIX and 28-day mortality was identified specifically within the CHF (*p* < 0.001), hypertension (*p* = 0.027), and CCI (*p* = 0.015) subgroup. In contrast, no significant interaction effects were observed in the other subgroups (*p* > 0.05). Furthermore, subgroup analyses based on different treatment strategies revealed a significant interaction effect only in the continuous renal replacement therapy (CRRT) subgroup (*p* = 0.042), while no notable interactions were observed in the other treatment subgroups (Figure 3(A)–(B)).

### E-value analysis for unmeasured confounding

**Figure 4.**
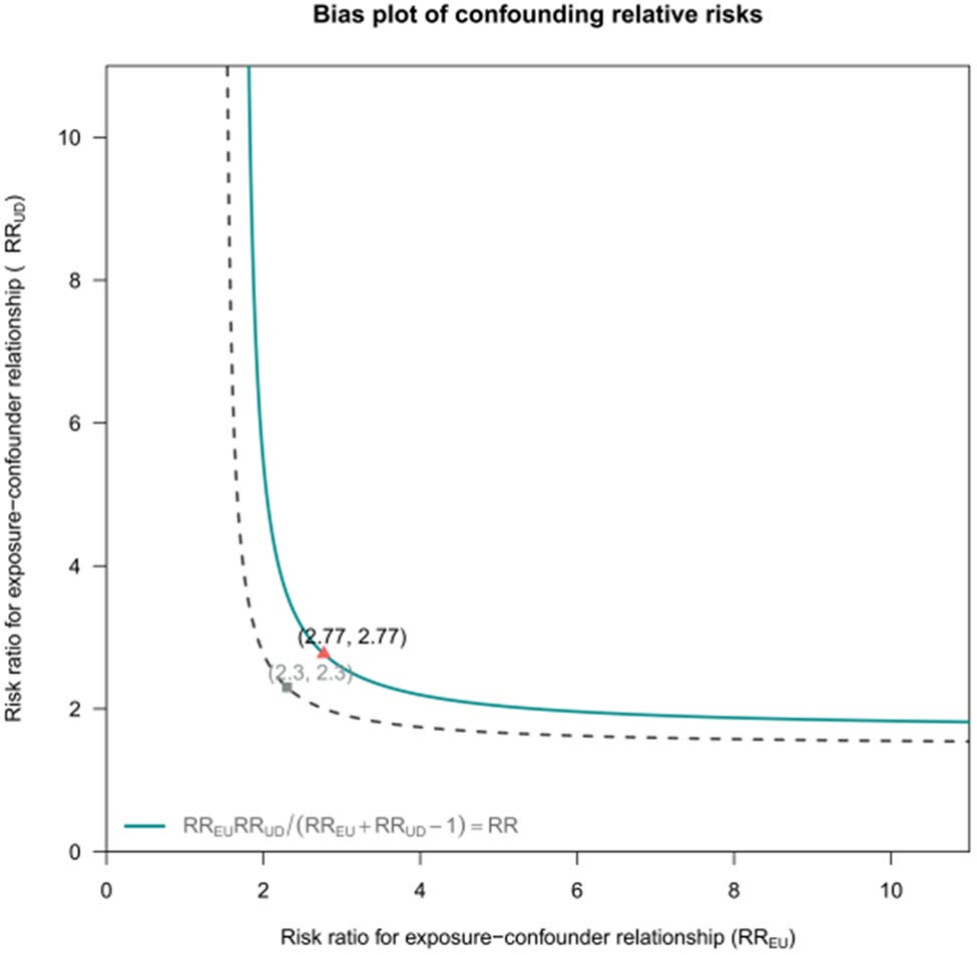
E-value analysis for unmeasured confounding.

Figure 4 shows the confounding-bias plot for the exposure-confounder relationship. The diagonal line represents the threshold where unmeasured confounding could significantly alter the observed association. Points beyond this line, like (2.77, 2.77), indicate the risk ratios needed for an unmeasured confounder to negate the observed EASIX–mortality effect. The point (2.77, 2.77) suggests that an unmeasured confounder would need to increase both the likelihood of high EASIX and 28-day mortality by 2.77 times to nullify the observed hazard ratio. The distance of potential confounders from the diagonal indicates that such a strong confounder is unlikely, reinforcing the robustness of our findings against residual confounding.

### Sensitivity analysis

**Figure 5.**
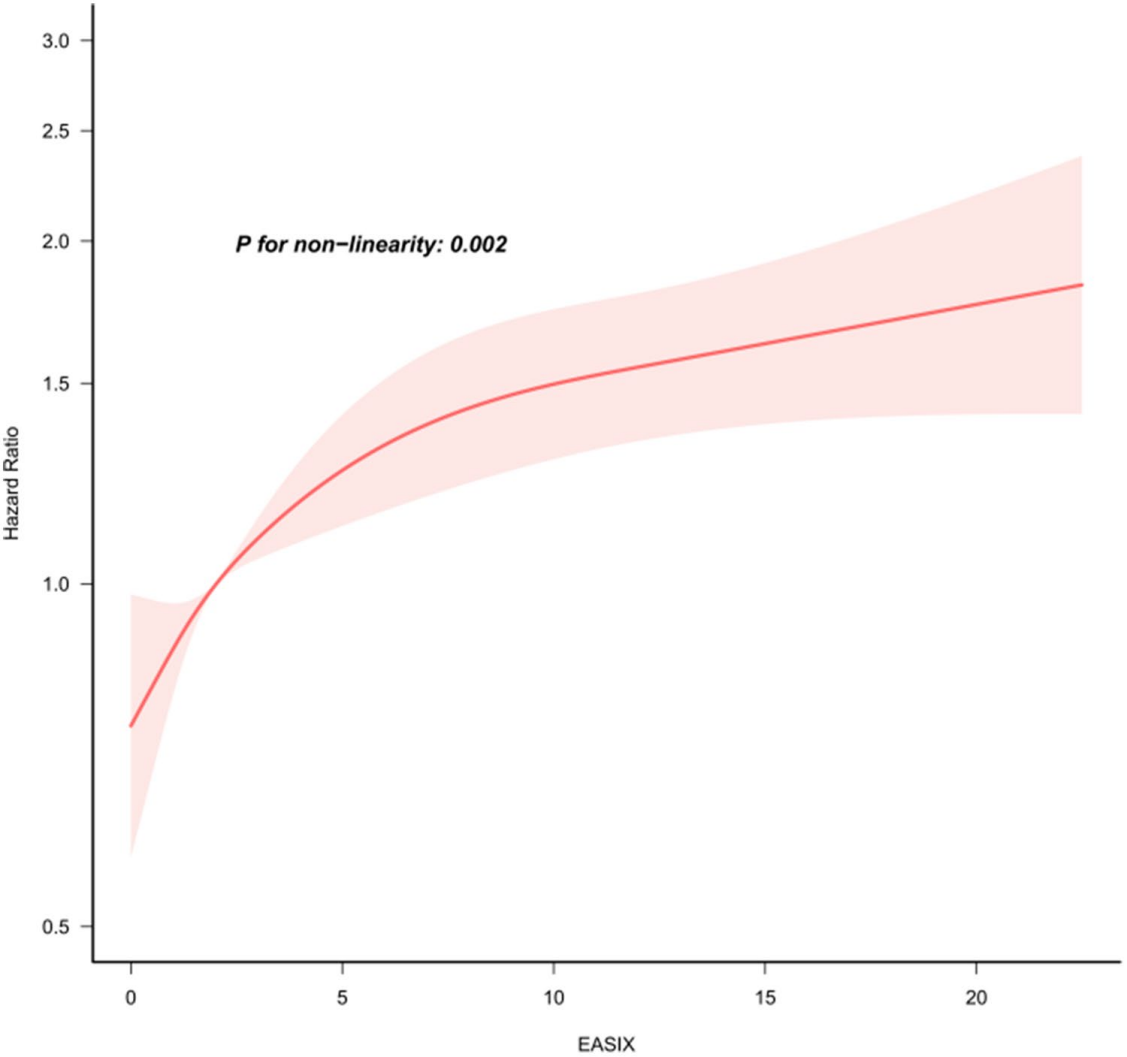
Curve fitting of the EASIX and 28-day mortality in patients with Elder AKI. Note: The data were adjusted for all potential confounding variables.

After treating EASIX as a continuous variable, the results of the Cox regression analysis exhibited a consistent trend with the tertile analysis, indicating that higher EASIX scores were associated with an increased risk of death in the ICU within 28 days (Supplementary Table 4). Subsequently, we employed a dose-response model to investigate the nonlinear relationship between EASIX and 28-day mortality, yielding a significant result (nonlinear test *p* = 0.002) (Figure 5). Furthermore, a threshold effect analysis demonstrated that the risk of mortality continued to rise with increasing EASIX levels up to a threshold of 174.855 (Slope1 = 1.0034, *p* < 0.001). Beyond this threshold, the risk curve plateaued, and statistical significance was lost (Slope2 = 1.00, *p* = 0.971) (Table 3). Finally, we conducted a comparative analysis of clinical outcomes between patients with AKI and those with end-stage renal disease (ESRD), revealing a higher mortality rate in the ESRD cohort (Supplementary Figure 1).

**Table 3.** Threshold effect analysis.

Item	HR (95%CI)	*p* value
	174.855 (167.759 − 181.952)	NA_character_
Slope1	1.0034 (1.002 − 1.0049)	**< 0.001**
Slope2	1 (0.9992 − 1.0008)	0.9709
Likelihood Ratio test		**<0.001**

Note: The data were adjusted for all potential confounding variables.

## Discussion

We enrolled in a cohort of 12,122 elderly patients with AKI to conduct a comprehensive evaluation of the EASIX across the entire disease continuum, from the onset to adverse outcomes. Initially, logistic regression analysis demonstrated a stepwise increase in AKI incidence corresponding to each EASIX tertile. The highest tertile was associated with an odds ratio (OR) of 1.65 (*p* < 0.001), suggesting that elevated EASIX serves not only as a prognostic marker but also as an early indicator of AKI development. Subsequently, Cox proportional hazards models indicated a graded increase in 28 days, ICU, and in-hospital mortality rates. The highest tertile exhibited a hazard ratio (HR) of 1.73 for 28-day mortality (*p* < 0.001), an effect that remained robust even after excluding the renal component of the SOFA score. Kaplan–Meier survival curves demonstrated a significant decline in survival probabilities with increasing EASIX values (log-rank *p* < 0.001). Further dose-response and threshold analyses revealed an inflection point at approximately 175. Below this threshold, the risk of mortality increased sharply, with a hazard ratio slope of 1.0034 per unit (*p* < 0.001). Beyond this point, the risk curve plateaued, indicating a clinically actionable range. Subgroup interaction analysis demonstrated that the association between EASIX and mortality was significantly amplified in the subgroups with CHF, hypertension, and a high CCI (interaction *p* < 0.001). Both the hypertension and high CCI subgroups identified EASIX as a risk factor. Importantly, within the CHF subgroup, individuals with CHF exhibited a higher mortality risk compared to those without CHF. This increased risk may be attributed to several factors: Firstly, CHF patients more frequently underwent intensive interventions such as mechanical ventilation, CRRT, vasopressor administration, diuretics, ACEI, and statins (Supplementary Figure 2), which could potentially improve prognosis. Secondly, patients with CHF, characterized by a persistent state of reduced cardiac output, may have developed renal compensatory mechanisms, including the activation of the renin–angiotensin system and the regulation of sodium and water retention, to adapt to diminished perfusion, thereby mitigating the risk of acute decompensation. Conversely, the renal systems of non-CHF patients may exhibit heightened sensitivity to acute hemodynamic alterations, rendering them more susceptible to AKI [22–24]. Thirdly, CHF patients often experience chronic low-grade inflammation and oxidative stress; however, the prolonged course of the disease may lead to the upregulation of endogenous antioxidant pathways, such as Sestrin2, which can alleviate organ damage [25]. In contrast, non-CHF patients may undergo more severe systemic inflammatory responses following AKI, potentially precipitating multi-organ failure [26,27]. Furthermore, CHF-associated microRNAs, such as miR-26, may modulate cardiorenal interactions by influencing fibrosis pathways [28]. In addition, subgroup analysis showed an interaction with CRRT (*p* = 0.042), indicating that in patients not on CRRT, a higher EASIX index correlates with the increased 28-day mortality risk. These findings suggest that early identification of high-EASIX patients and timely initiation of CRRT may improve outcomes. Other treatment results indicate stability. Finally, the E-value sensitivity analysis showed that an unmeasured confounder would need a relative risk of 2.77 for both exposure–outcome and exposure–confounder links to negate our findings, highlighting their robustness. Then, a sensitivity analysis was conducted to compare the mortality rates between individuals with AKI and those with ESRD. The findings indicated that individuals with ESRD exhibited higher 28-day mortality rates, as well as increased mortality within the intensive care unit (ICU) and during hospitalization [29,30]. Overall, EASIX is a comprehensive tool for early detection and risk assessment in elderly AKI patients, potentially guiding future personalized treatments.

The EASIX is determined by the formula: creation multiplied by lactate dehydrogenase, divided by platelets. As a laboratory prognostic marker, it serves as an indicator of endothelial activation and stress. In the context of AKI, an elevated EASIX value is associated with the adverse outcomes, including ICU admission and mortality. This association may be attributed to endothelial cell damage, heightened oxidative stress (e.g., increased NOX4 expression), and inflammatory responses [31]. Specifically, endothelial activation results in increased levels of inflammatory markers such as tumor necrosis factor-alpha (TNFα), interleukin-1 (IL1), intercellular adhesion molecule-1 (ICAM-1), and vascular cell adhesion molecule-1 (VCAM-1). These markers not only intensify renal tissue damage but also impede recovery from AKI by inducing microvascular dysfunction [32]. For instance, in the context of CAR-T cell therapy, EASIX is linked to complications, although the underlying biological pathways remain incompletely understood. Following AKI, heightened inflammatory responses, such as macrophage infiltration, can lead to tubular injury and subsequent fibrosis. Inhibition of microRNA-21 (miR-21) has been shown to mitigate macrophage-induced damage and facilitate the repair process by elevating heparan sulfate (HS) levels [33]. In gerontological models, the upregulation of inflammatory markers, including tumor necrosis factor-alpha (TNFα) and interleukin-1 (IL-1), has been implicated in the induction of apoptosis in tubular epithelial cells and the development of interstitial fibrosis. Furthermore, the activation of the complement system, exemplified by the deposition of complement components C3d and C5b-9 within the renal tubules, is a critical factor in the pathogenesis of rhabdomyolysis-induced AKI, thereby exacerbating the clinical prognosis [34,35]. Tubular injury, a fundamental pathological characteristic of AKI, is facilitated by the dysregulation of the actin cytoskeleton, which adversely impacts the function and recovery of tubular epithelial cells. The progression of fibrosis is associated with the accumulation of uremic toxins, such as indoxyl sulfate (IS), which enhances collagen expression and activates matrix metalloproteinase-9, thereby contributing to the transition from AKI to chronic kidney disease (CKD) [36–38]. Exogenous interventions, such as the inhibition of miR-21, or the reduction of oxidative stress through NOX4 inhibition, have the potential to alleviate damage and enhance recovery processes. For instance, treatment with AVA has been shown to lower inflammatory markers and decrease age-related adverse events associated with treatment, such as AKI. These repair mechanisms offer therapeutic strategy potential for elderly patients with AKI and need to be validated in clinical trials [39].

The findings of this study bear significant implications for the clinical management of elderly patients experiencing AKI. Given the decline in physiological function and the prevalence of multiple comorbidities in this population, the rapid and complex progression of their condition is of critical importance. EASIX emerges as a potential prognostic biomarker for the early identification of high-risk patients, particularly those with concurrent cardiovascular disease. Through dynamic monitoring of EASIX levels, clinicians can more accurately discern which elderly patients are at heightened risk of developing AKI or experiencing poor prognostic outcomes, thereby facilitating the timely implementation of targeted interventions. For instance, in elderly patients exhibiting elevated EASIX levels, it may be prudent to consider more frequent monitoring of renal function, optimization of hemodynamic support, early initiation of RRT, or adjustment of medication dosages to mitigate the incidence and mortality associated with AKI. Furthermore, the EASIX threshold of 175 may function as a potential ‘clinical intervention trigger point,’ as within this range, the patient’s 28-day mortality risk escalates with increasing EASIX levels (HR 1.69). This suggests that enhanced management strategies should be implemented as this threshold is neared. Such strategies include intensified monitoring of urine output and creatinine levels, optimization of volume status and hemodynamics, early evaluation of the need for RRT, and consideration of antioxidants or endothelial protective agents to delay or prevent the progression of endothelial injury.

Although this study provides compelling evidence of the association between EASIX and the prognosis of elderly patients with AKI, it is not without limitations. Firstly, while the calculation of the E value has been performed, the retrospective and single-center design of the MIMIC-IV study may constrain causal inferences and limit the generalizability of the findings. Secondly, the absence of direct indicators of endothelial function, such as ICAM-1, VCAM-1, VEGF, and nitric oxide, undermines the biological rationale. Thirdly, although an EASIX threshold of 175 may indicate the need for enhanced monitoring or early RRT, this threshold necessitates external validation prior to clinical implementation. Future investigations should be designed as multicenter, prospective, large-scale cohort studies to systematically gather direct indicators of endothelial function, such as ICAM-1, VCAM-1, VEGF, and nitric oxide [40]. This approach will elucidate the biological underpinnings of EASIX and enhance the understanding of the causal relationship between EASIX and clinical outcomes. Furthermore, external validation across geographically diverse populations is essential to verify the universality and safety of the 175 thresholds. Ultimately, these findings should be integrated into risk-stratified intervention pathways for AKI in elderly patients.

## Conclusion

An elevated EASIX is significantly correlated with the onset of AKI and unfavorable short-term outcomes, such as heightened mortality rates. This underscores the potential utility of EASIX as a prognostic biomarker for the early identification and risk stratification of elderly patients with AKI.

## Supplementary Material

Supplementary Table 4.docx

Supplementary Figure 2.tif

Supplementary Figure 1.tif

Supplementary Table 1.docx

Supplementary Table 3.docx

Manuscript_Figures_Tables_SupplFiles_KZou.zip.zip

Supplementary Table 2.docx

## Data Availability

The datasets generated and/or analyzed in the present study can be accessed through the MIMIC-IV database, available at https://mimic.physionet.org/iv/.
