# Supplementary material for: The association between endothelial activation and stress Index and the development and prognosis of acute kidney injury in elderly patients with critical illness
Source: Ren Fail. 2025 Nov 4;47(1):2577174. doi: 10.1080/0886022X.2025.2577174 (PMC12587800; doi:10.1080/0886022X.2025.2577174)
Supplement: Supplementary Table 4.docx [file IRNF_A_2577174_SM7696.docx]

**Supplementary Table 4. Multivariable Cox Regression Analysis of Primary Clinical Outcomes (EASIX as a continuous variable).**

|  | Unadjusted | |  | Model 1 | |  | Model 2 | |
| --- | --- | --- | --- | --- | --- | --- | --- | --- |
| Variable | HR（95% CI） | p value |  | HR（95% CI） | p value |  | HR（95% CI） | p value |
| **28-day mortality** | 1.0002 (1.0001~1.0002) | **<0.001** |  | 1.0002 (1.0002~1.0003) | **<0.001** |  | 1.0001 (1~1.0002) | **0.008** |
| **In-icu mortality** | 1.0002 (1.0001~1.0002) | **<0.001** |  | 1.0002 (1.0002~1.0002) | **<0.001** |  | 1.0001 (1.0001~1.0002) | **<0.001** |
| **In-hospital mortality** | 1.0001 (1~1.0002) | 0.093 |  | 1.0001 (1~1.0003) | **0.017** |  | 1 (0.9999~1.0002) | 0.813 |

**Unadjusted**: Crude model without covariate adjustment.

**Model 1**: Adjusted for age, sex, and ethnicity.

**Model 2**: Adjusted for age, sex, ethnicity, weight, vital signs (heart rate, respiration rate, SpO₂, mean blood pressure), scoring systems (SOFA, CCI, APSIII, OASIS), comorbidities (hypertension, diabetes, liver disease, myocardial infarct, congestive heart failure, cerebrovascular disease, chronic pulmonary disease, malignant cancer, sepsis), laboratory results (WBC, RBC, hemoglobin, sodium, potassium, calcium, chloride, glucose, total bilirubin, BUN), and interventions (ventilation, CRRT, vasopressin, diuretic, ACEI, Stains).
P-values less than 0.05 are expressed in bold.
