# Supplementary figures and images for: The association between endothelial activation and stress Index and the development and prognosis of acute kidney injury in elderly patients with critical illness
Source: Ren Fail. 2025 Nov 4;47(1):2577174. doi: 10.1080/0886022X.2025.2577174 (PMC12587800; doi:10.1080/0886022X.2025.2577174)

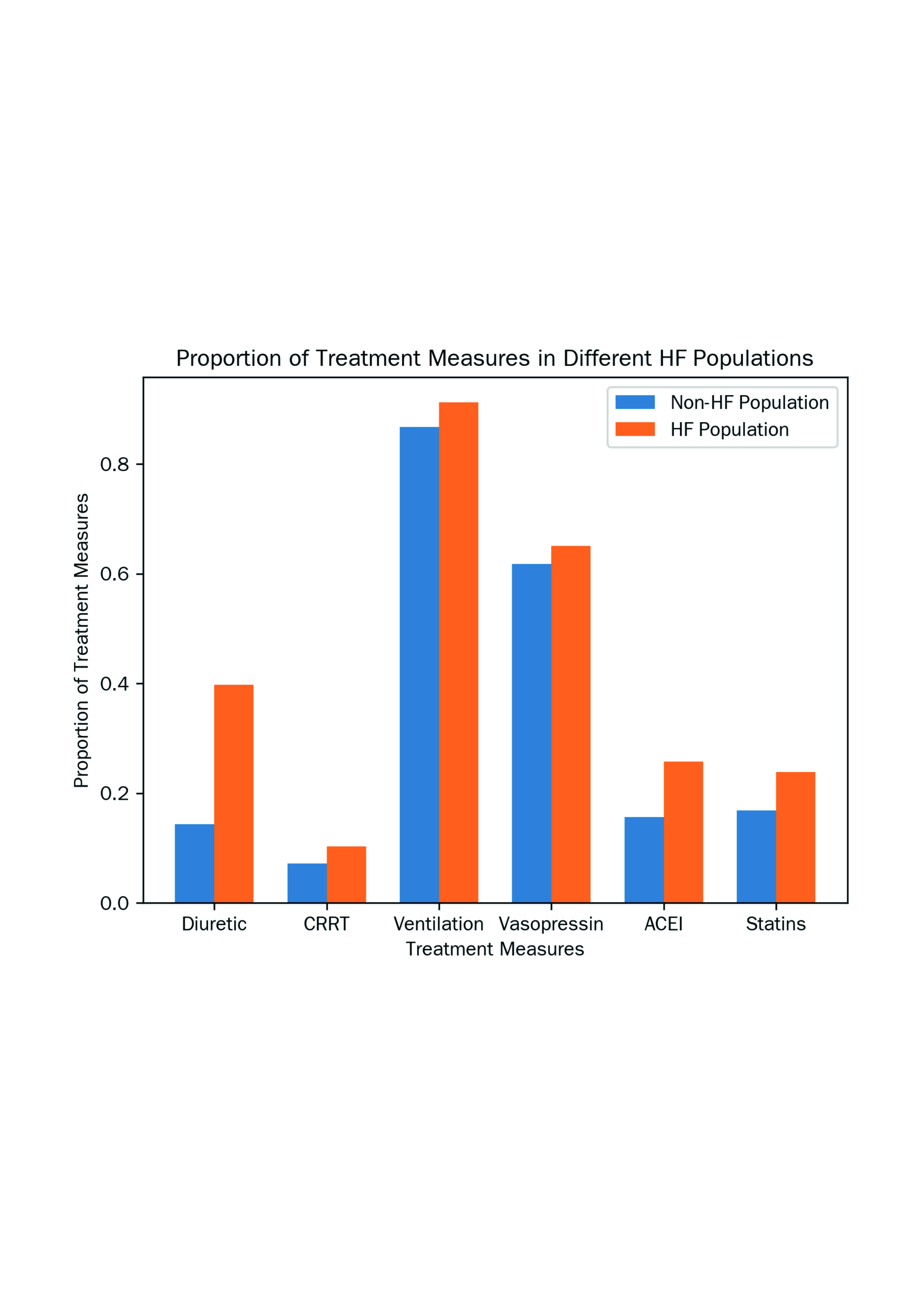

Supplement: Supplementary Figure 2.tif [file IRNF_A_2577174_SM7695.tif]

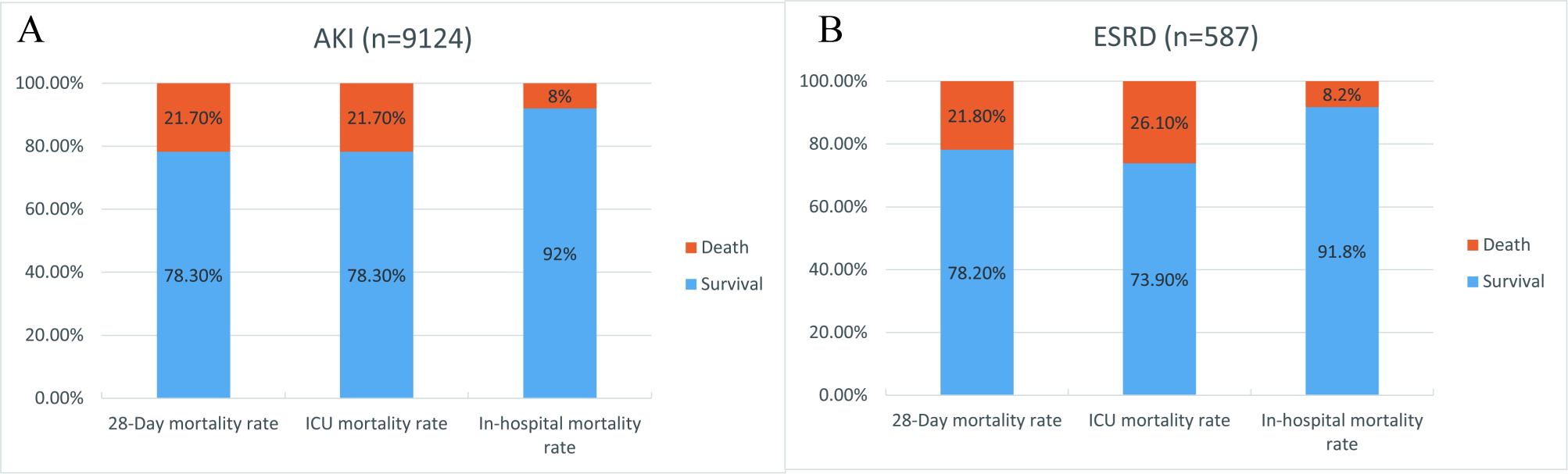

Supplement: Supplementary Figure 1.tif [file IRNF_A_2577174_SM7694.tif]

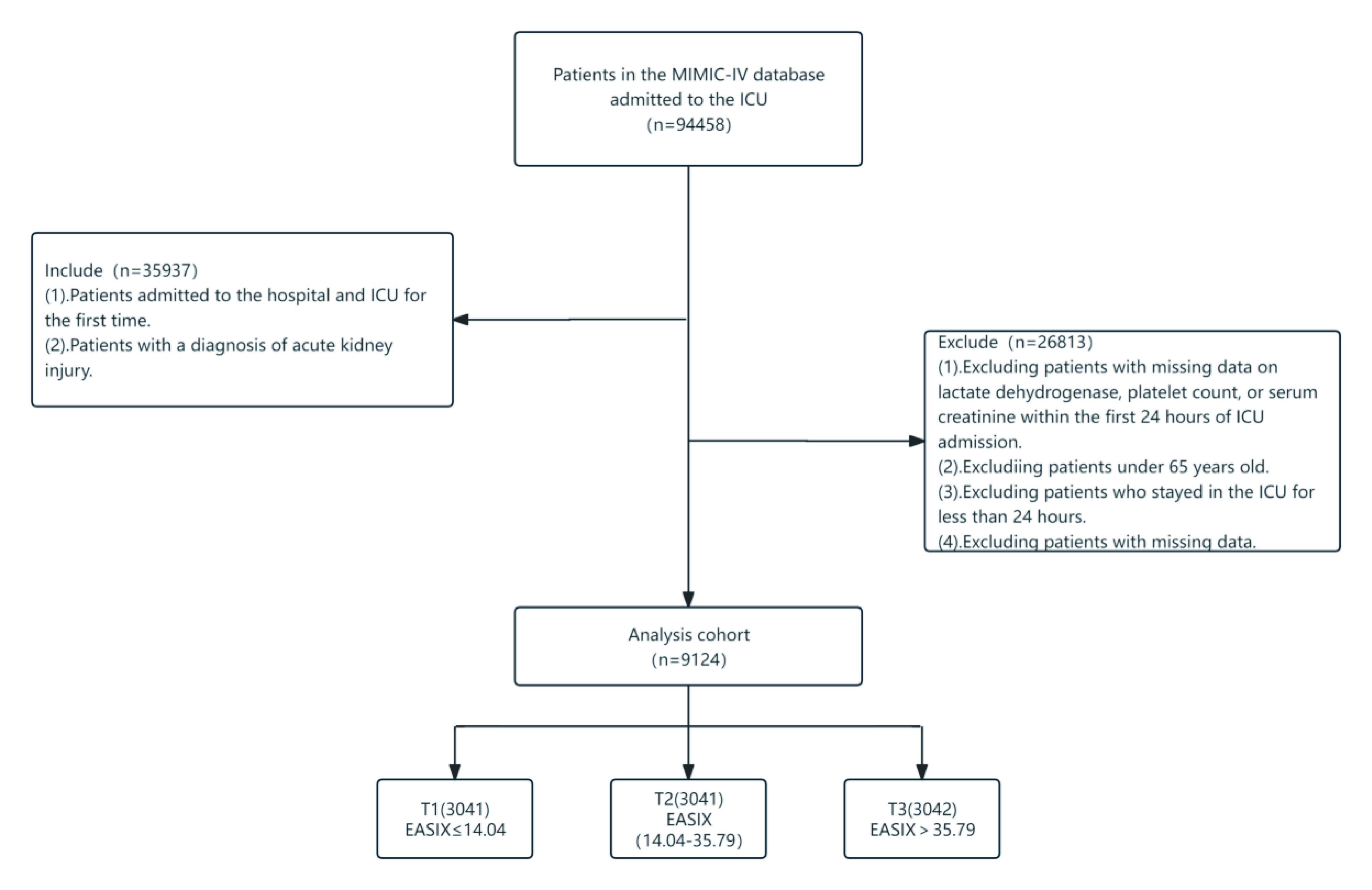

Supplement: Manuscript_Figures_Tables_SupplFiles_KZou.zip.zip [file IRNF_A_2577174_SM7691.zip › figures, tables and supplementary files/Figure1.tif]

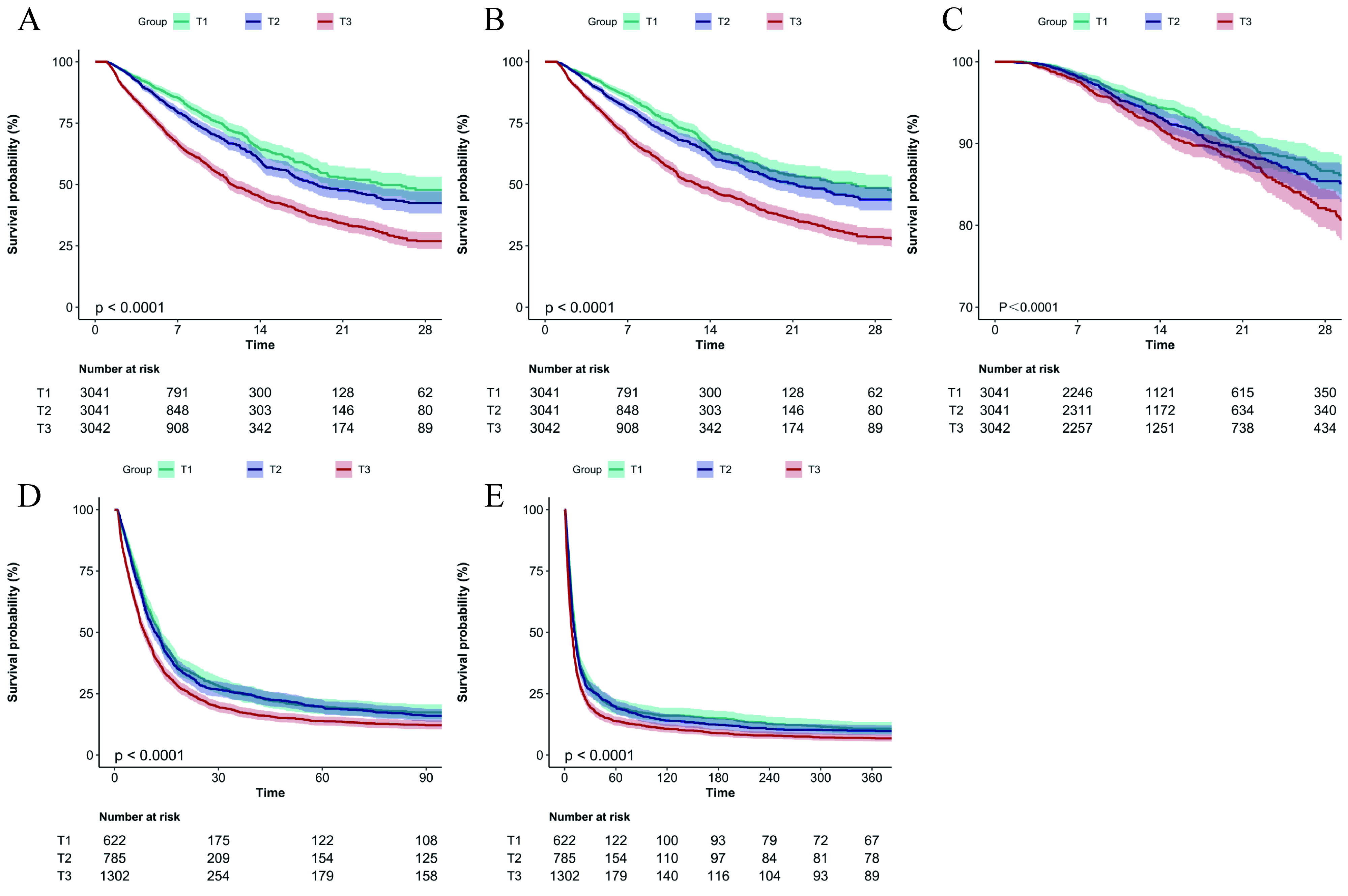

Supplement: Manuscript_Figures_Tables_SupplFiles_KZou.zip.zip [file IRNF_A_2577174_SM7691.zip › figures, tables and supplementary files/Figure2.tif]

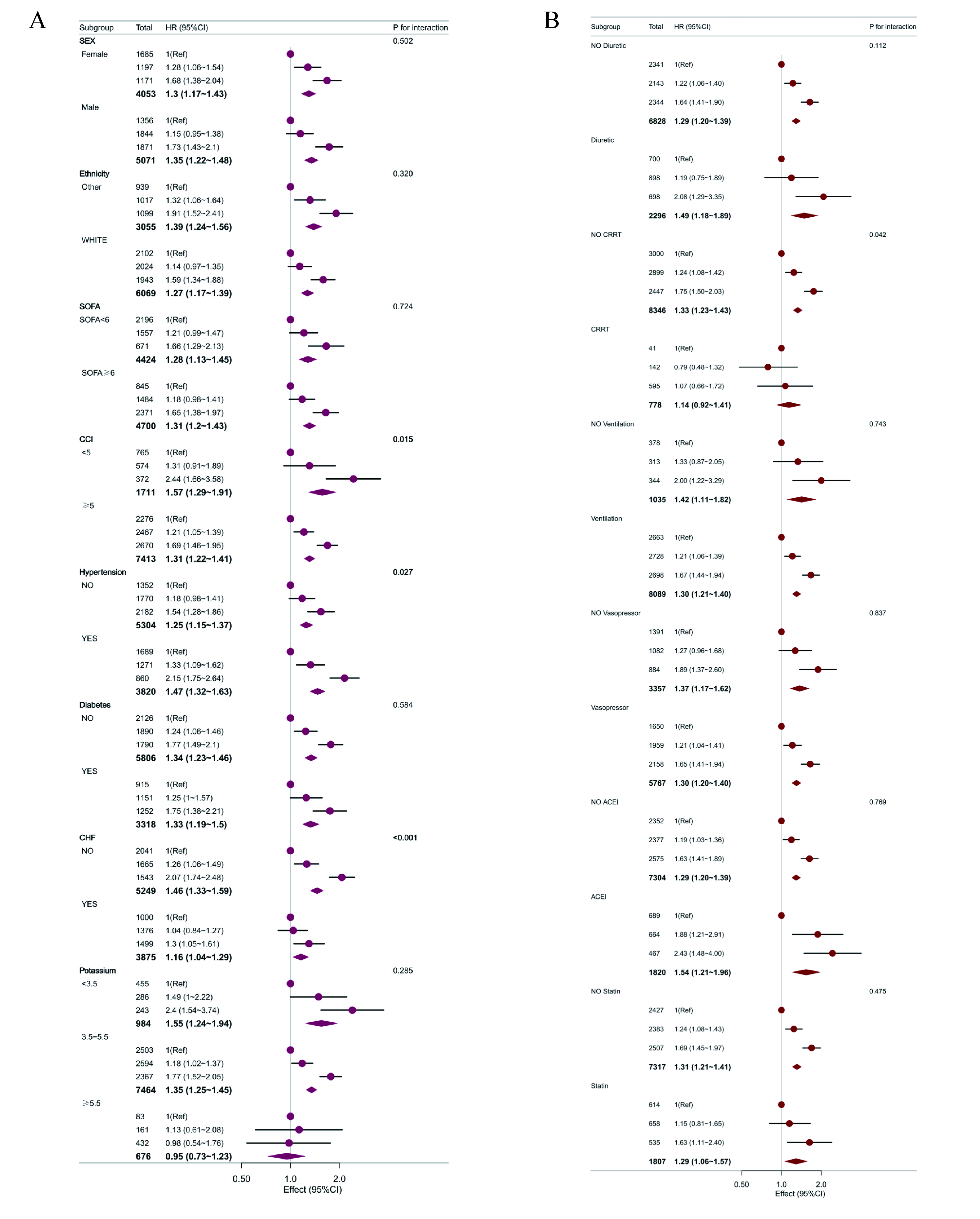

Supplement: Manuscript_Figures_Tables_SupplFiles_KZou.zip.zip [file IRNF_A_2577174_SM7691.zip › figures, tables and supplementary files/Figure3.tif]

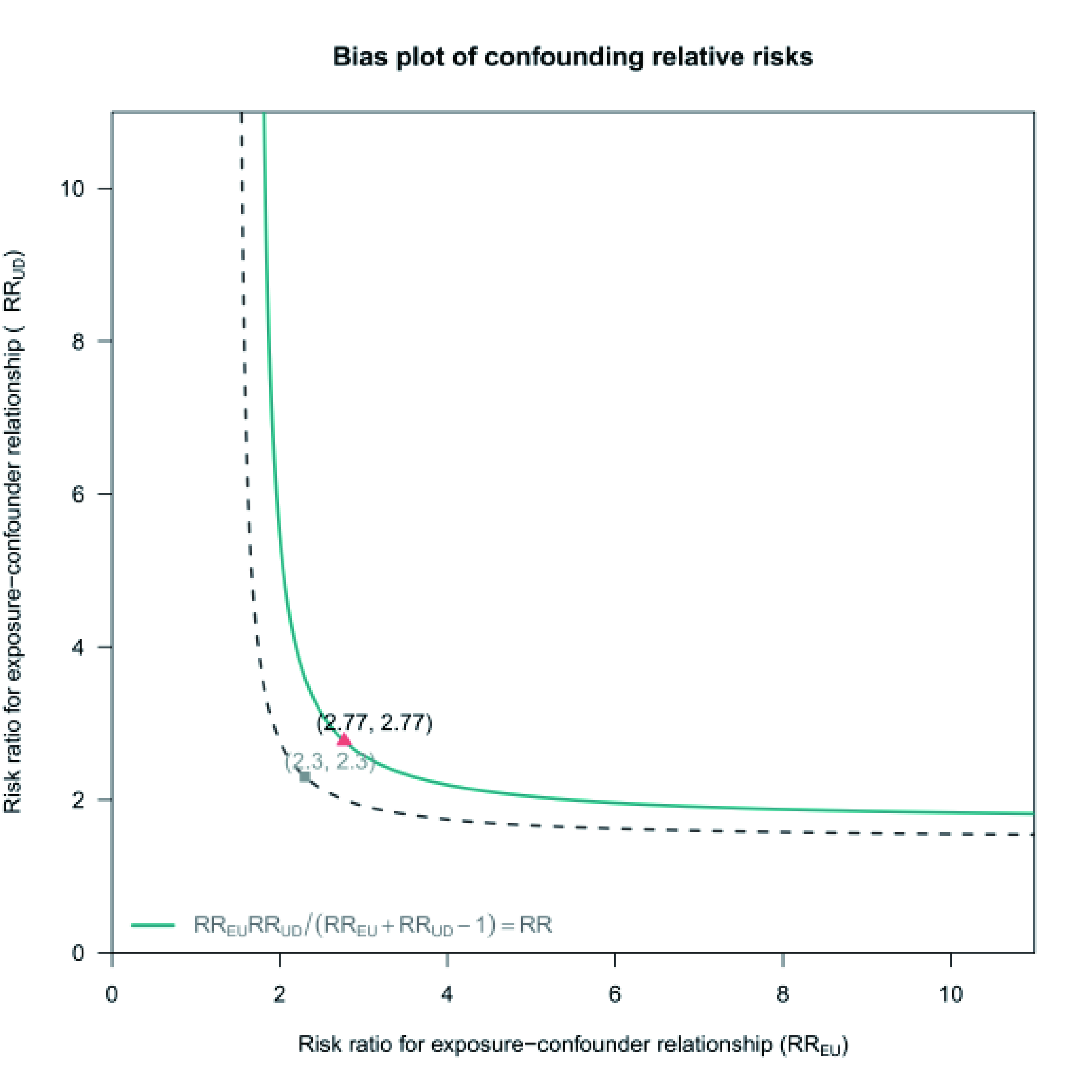

Supplement: Manuscript_Figures_Tables_SupplFiles_KZou.zip.zip [file IRNF_A_2577174_SM7691.zip › figures, tables and supplementary files/Figure4.tif]

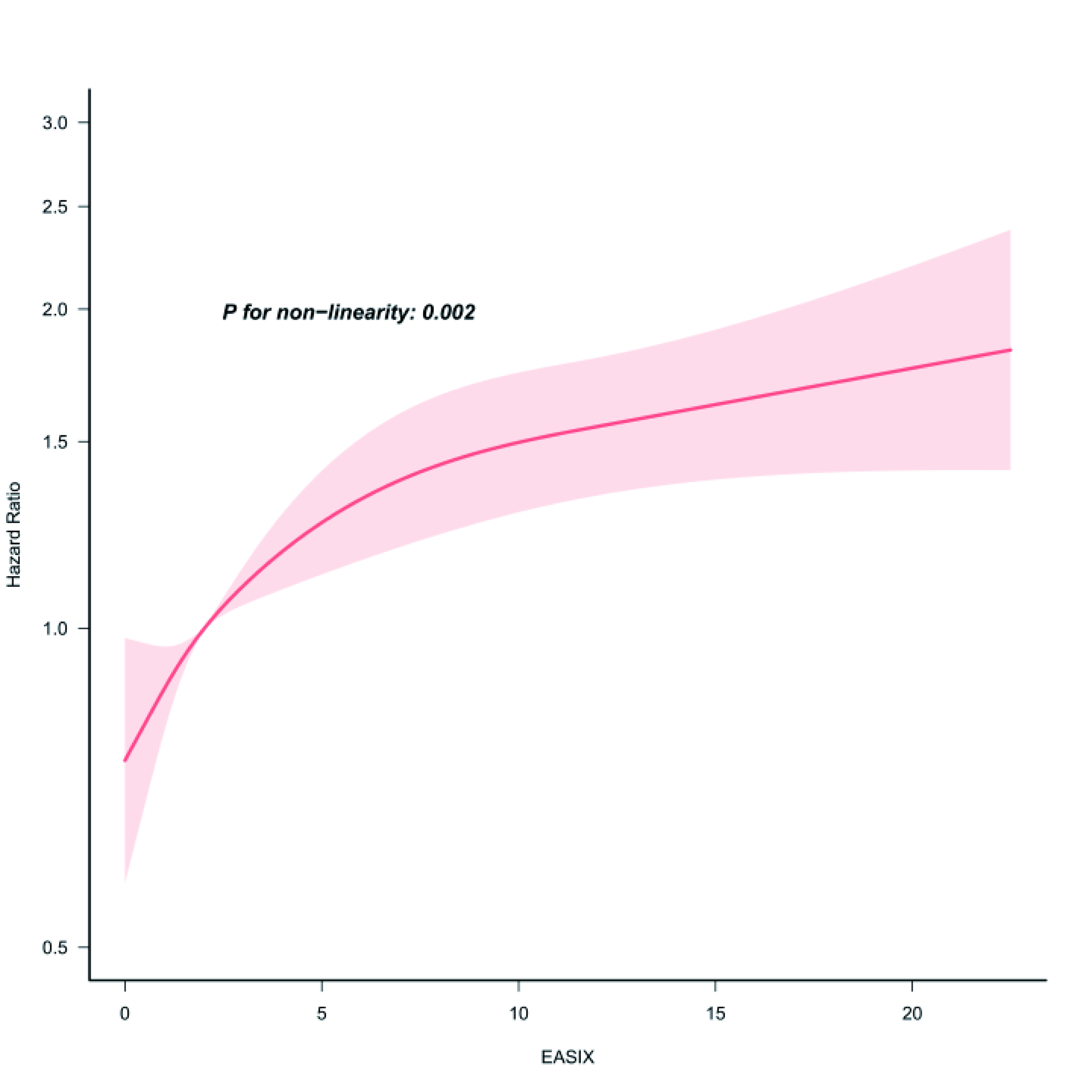

Supplement: Manuscript_Figures_Tables_SupplFiles_KZou.zip.zip [file IRNF_A_2577174_SM7691.zip › figures, tables and supplementary files/Figure5.tif]

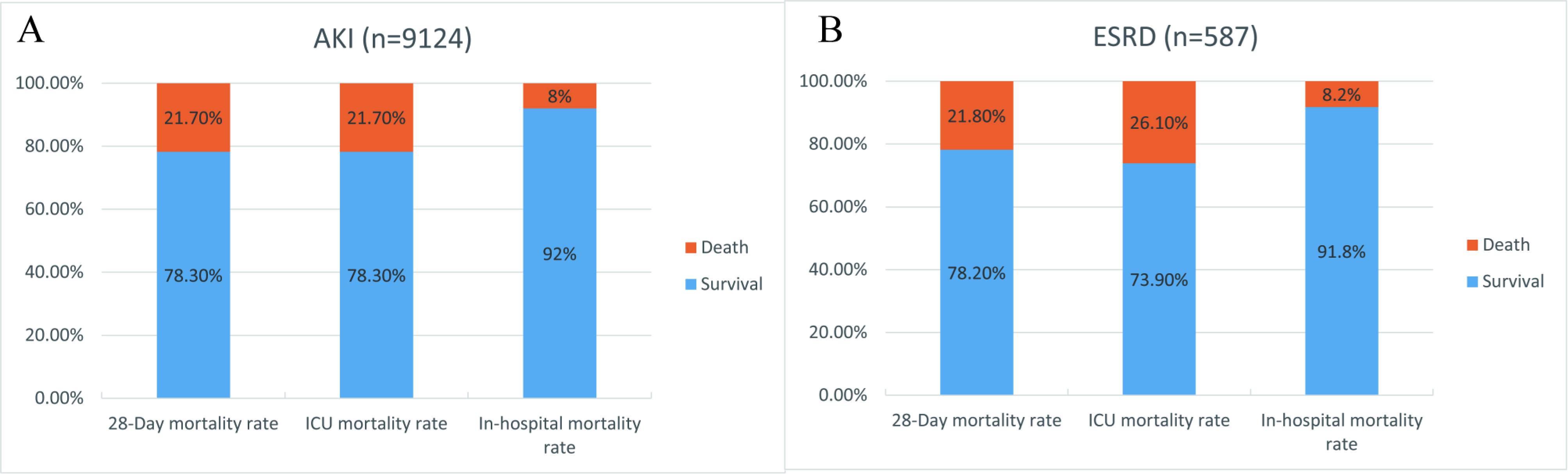

Supplement: Manuscript_Figures_Tables_SupplFiles_KZou.zip.zip [file IRNF_A_2577174_SM7691.zip › figures, tables and supplementary files/Supplementary Figure 1.tif]

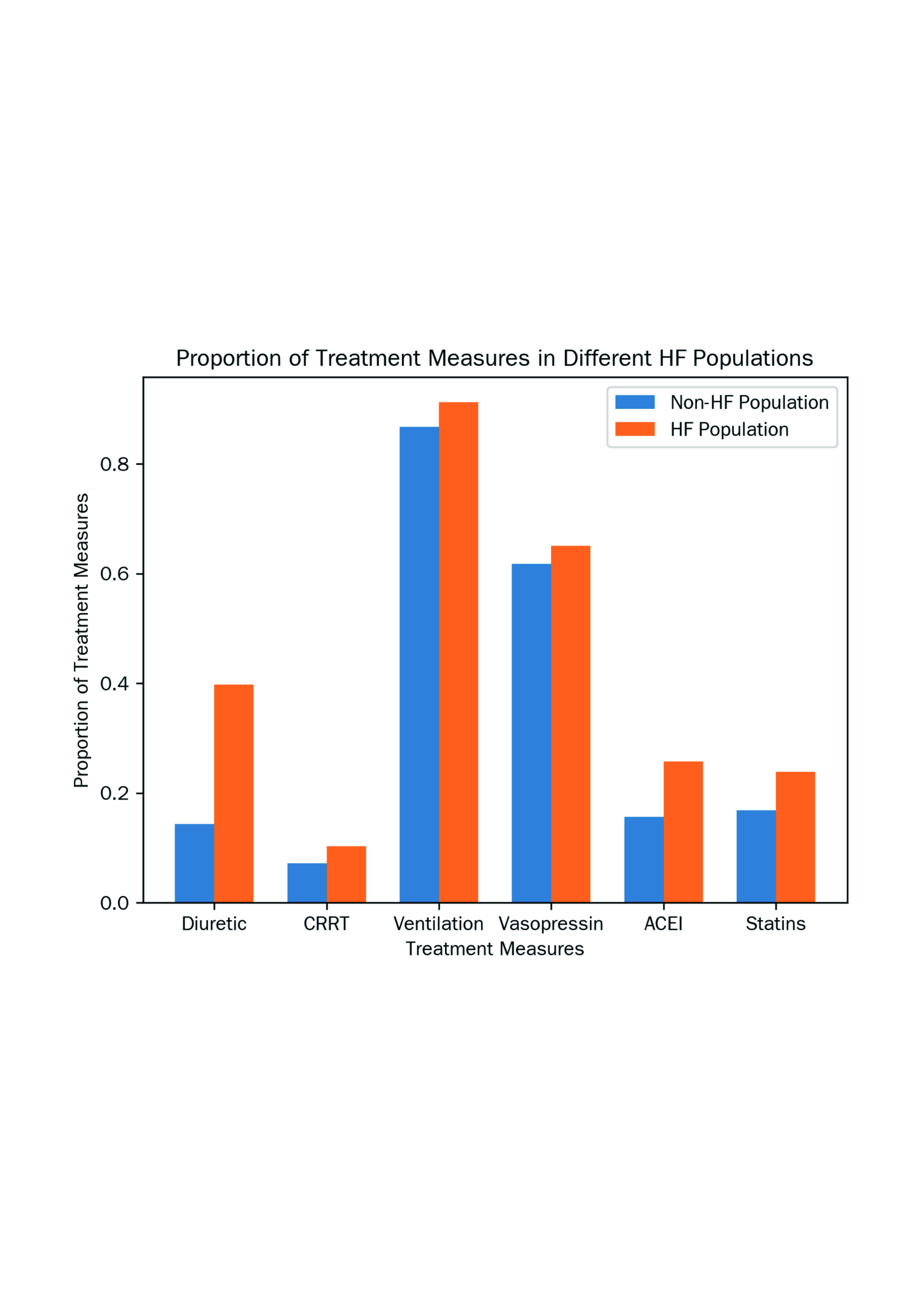

Supplement: Manuscript_Figures_Tables_SupplFiles_KZou.zip.zip [file IRNF_A_2577174_SM7691.zip › figures, tables and supplementary files/Supplementary Figure 2.tif]
