# Supplementary material for: The association between endothelial activation and stress Index and the development and prognosis of acute kidney injury in elderly patients with critical illness
Source: Ren Fail. 2025 Nov 4;47(1):2577174. doi: 10.1080/0886022X.2025.2577174 (PMC12587800; doi:10.1080/0886022X.2025.2577174)
Supplement: Supplementary Table 1.docx [file IRNF_A_2577174_SM7693.docx]

**Supplementary Table 1. Baseline characteristics of participants.**

| Variables | Total  (n = 12122) | **Endothelial Activation and Stress Index** | | | ***P*** value |
| --- | --- | --- | --- | --- | --- |
|  |  | T1 | T2 | T3 |  |
|  |  | (n = 4041) | (n = 4040) | (n = 4041) |  |
| **Age (years)** | 77.1 ± 8.0 | 77.0 ± 8.0 | 77.7 ± 8.1 | 76.8 ± 7.9 | **< 0.001** |
| **Sex** (%) |  |  |  |  | **< 0.001** |
| F | 5480 (45.2) | 2278 (56.4) | 1659 (41.1) | 1543 (38.2) |  |
| M | 6642 (54.8) | 1763 (43.6) | 2381 (58.9) | 2498 (61.8) |  |
| **Ethnicity (%)** |  |  |  |  | **< 0.001** |
| OTHER | 4018 (33.1) | 1229 (30.4) | 1339 (33.1) | 1450 (35.9) |  |
| WHITE | 8104 (66.9) | 2812 (69.6) | 2701 (66.9) | 2591 (64.1) |  |
| **Weight (Kg)** | 79.3 ± 21.4 | 75.0 ± 20.8 | 80.6 ± 21.2 | 82.2 ± 21.4 | **< 0.001** |
| **Vital signs** |  |  |  |  |  |
| Heart rate (bpm) | 88.6 ± 20.8 | 88.4 ± 20.8 | 87.7 ± 20.5 | 89.8 ± 21.1 | **< 0.001** |
| Respiration (bpm) | 19.9 ± 6.4 | 19.7 ± 6.2 | 19.6 ± 6.3 | 20.5 ± 6.5 | **< 0.001** |
| Spo2 (%) | 96.6 ± 9.0 | 96.7 ± 3.7 | 96.7 ± 4.2 | 96.4 ± 14.6 | 0.295 |
| MBP (mmHg) | 81.5 ± 19.1 | 84.0 ± 18.9 | 81.3 ± 18.9 | 79.2 ± 19.2 | **< 0.001** |
| **Scoring system, points** |  |  |  |  |  |
| SOFA | 5.6 ± 3.5 | 3.7 ± 2.6 | 5.2 ± 2.9 | 7.9 ± 3.5 | **< 0.001** |
| CCI | 6.8 ± 2.6 | 6.1 ± 2.4 | 6.7 ± 2.5 | 7.5 ± 2.6 | **< 0.001** |
| Apsiii | 50.7 ± 20.7 | 42.7 ± 16.8 | 48.4 ± 18.8 | 61.2 ± 21.9 | **< 0.001** |
| Oasis | 33.9 ± 8.5 | 32.7 ± 7.9 | 33.5 ± 8.2 | 35.6 ± 9.0 | **< 0.001** |
| **Comorbidities** |  |  |  |  |  |
| Hypertension (%) | 5234 (43.2) | 2271 (56.2) | 1753 (43.4) | 1210 (29.9) | **< 0.001** |
| Diabetes (%) | 4239 (35.0) | 1140 (28.2) | 1469 (36.4) | 1630 (40.3) | **< 0.001** |
| Liver Disease (%) | 7833 (64.6) | 2739 (67.8) | 2637 (65.3) | 2457 (60.8) | **< 0.001** |
| Myocardial Infarct (%) | 1567 (12.9) | 278 (6.9) | 509 (12.6) | 780 (19.3) | **< 0.001** |
| Congestive Heart Failure (%) | 4760 (39.3) | 1123 (27.8) | 1690 (41.8) | 1947 (48.2) | **< 0.001** |
| Cerebrovascular Disease (%) | 2586 (21.3) | 918 (22.7) | 856 (21.2) | 812 (20.1) | **0.015** |
| Chronic Pulmonary Disease (%) | 1323 (10.9) | 433 (10.7) | 472 (11.7) | 418 (10.3) | 0.137 |
| Malignant Cancer (%) | 2623 (21.6) | 906 (22.4) | 879 (21.8) | 838 (20.7) | 0.180 |
| Sepsis (%) | 7628 (62.9) | 2120 (52.5) | 2525 (62.5) | 2983 (73.8) | **< 0.001** |
| **Laboratory results** |  |  |  |  |  |
| WBC (K/uL) | 13.2 ± 13.1 | 12.6 ± 7.5 | 12.8 ± 10.9 | 14.3 ± 18.4 | **< 0.001** |
| RBC (K/uL) | 3.5 ± 0.8 | 3.6 ± 0.7 | 3.5 ± 0.8 | 3.3 ± 0.8 | **< 0.001** |
| Hemoglobin (g/dL） | 10.4 ± 2.2 | 10.8 ± 2.1 | 10.5 ± 2.2 | 10.0 ± 2.3 | **< 0.001** |
| Sodium (mEq/L) | 138.3 ± 5.8 | 137.9 ± 5.6 | 138.7 ± 5.4 | 138.2 ± 6.2 | **< 0.001** |
| Potassium (mEq/L) | 4.2 ± 0.8 | 4.1 ± 0.6 | 4.2 ± 0.7 | 4.5 ± 0.9 | **< 0.001** |
| Calciumtotal (mg/dL) | 8.3 ± 0.9 | 8.4 ± 0.8 | 8.4 ± 0.8 | 8.3 ± 0.9 | **< 0.001** |
| Chloride (mEq/L) | 103.7 ± 6.9 | 103.2 ± 6.6 | 104.4 ± 6.5 | 103.4 ± 7.5 | **< 0.001** |
| Glucose (mg/dL) | 150.8 ± 75.3 | 139.9 ± 58.3 | 152.9 ± 78.4 | 159.6 ± 85.1 | **< 0.001** |
| Total Bilirubin (mg/dL) | 1.3 ± 2.7 | 0.9 ± 1.7 | 1.2 ± 2.4 | 1.8 ± 3.6 | **< 0.001** |
| BUN (mg/dL) | 32.7 ± 25.0 | 20.3 ± 12.1 | 29.6 ± 18.7 | 48.1 ± 31.3 | **< 0.001** |
| **Interventions** |  |  |  |  |  |
| Ventilation (%) | 10031 (82.8) | 3181 (78.7) | 3389 (83.9) | 3461 (85.6) | **< 0.001** |
| CRRT (%) | 783 (6.5) | 32 (0.8) | 111 (2.7) | 640 (15.8) | **< 0.001** |
| Vasopressin (%) | 6676 (55.1) | 1779 (44) | 2241 (55.5) | 2656 (65.7) | **< 0.001** |
| Diuretic (%) | 2884 (23.8) | 778 (19.3) | 1131 (28) | 975 (24.1) | **< 0.001** |
| **Hospital stays** | 14.4 ± 13.8 | 13.4 ± 12.5 | 13.9 ± 12.9 | 15.8 ± 15.8 | **< 0.001** |
| **Icu stay** | 5.6 ± 6.8 | 5.0 ± 6.1 | 5.5 ± 7.0 | 6.3 ± 7.4 | **< 0.001** |
| **AKI** | 9124 (75.3) | 2646 (65.5) | 3028 (75) | 3450 (85.4) | **< 0.001** |
