# Supplementary material for: The association between endothelial activation and stress Index and the development and prognosis of acute kidney injury in elderly patients with critical illness
Source: Ren Fail. 2025 Nov 4;47(1):2577174. doi: 10.1080/0886022X.2025.2577174 (PMC12587800; doi:10.1080/0886022X.2025.2577174)
Supplement: Manuscript_Figures_Tables_SupplFiles_KZou.zip.zip [file IRNF_A_2577174_SM7691.zip › figures, tables and supplementary files/Table 1.docx]

**Table 1. Baseline characteristics of participants and outcome parameters.**

|  | Total  (n = 9124) | **EASIX** | | | ***P*** value |
| --- | --- | --- | --- | --- | --- |
| Variables |  | T1 | T2 | T3 |  |
|  |  | (n = 3041) | (n = 3041) | (n = 3042) |  |
| **Age (years)** | 77.2 ± 8.0 | 77.3 ± 8.1 | 77.6 ± 7.9 | 76.7 ± 7.9 | **< 0.001** |
| **Sex** (%) |  |  |  |  | **< 0.001** |
| F | 4053 (44.4) | 1685 (55.4) | 1197 (39.4) | 1171 (38.5) |  |
| M | 5071 (55.6) | 1356 (44.6) | 1844 (60.6) | 1871 (61.5) |  |
| **Ethnicity (%)** |  |  |  |  | **< 0.001** |
| OTHER | 3055 (33.5) | 939 (30.9) | 1017 (33.4) | 1099 (36.1) |  |
| WHITE | 6069 (66.5) | 2102 (69.1) | 2024 (66.6) | 1943 (63.9) |  |
| **Weight (Kg)** | 79.3 ± 21.4 | 75.0 ± 20.8 | 80.6 ± 21.2 | 82.2 ± 21.4 | **< 0.001** |
| **Vital signs** |  |  |  |  |  |
| Heart rate (bpm) | 89.1 ± 21.0 | 89.2 ± 20.8 | 88.2 ± 20.9 | 89.8 ± 21.1 | **0.010** |
| Respiration (bpm) | 20.0 ± 6.5 | 19.9 ± 6.2 | 19.7 ± 6.5 | 20.5 ± 6.6 | **< 0.001** |
| Spo2 (%) | 96.6 ± 10.2 | 96.7 ± 3.9 | 96.6 ± 5.1 | 96.4 ± 16.5 | 0.681 |
| MBP (mmHg) | 80.7 ± 19.3 | 82.8 ± 19.4 | 80.7 ± 18.7 | 78.6 ± 19.6 | **< 0.001** |
| **Scoring system, points** |  |  |  |  |  |
| SOFA | 6.2 ± 3.6 | 4.2 ± 2.8 | 5.9 ± 3.0 | 8.4 ± 3.5 | **< 0.001** |
| CCI | 6.9 ± 2.6 | 6.3 ± 2.5 | 6.8 ± 2.6 | 7.5 ± 2.6 | **< 0.001** |
| Apsiii | 53.8 ± 21.4 | 45.7 ± 17.8 | 51.5 ± 19.7 | 64.3 ± 22.3 | **< 0.001** |
| Oasis | 35.3 ± 8.5 | 34.5 ± 7.9 | 34.8 ± 8.3 | 36.7 ± 9.1 | **< 0.001** |
| **Comorbidities** |  |  |  |  |  |
| Hypertension (%) | 3820 (41.9) | 1689 (55.5) | 1271 (41.8) | 860 (28.3) | **< 0.001** |
| Diabetes (%) | 3318 (36.4) | 915 (30.1) | 1151 (37.8) | 1252 (41.2) | **< 0.001** |
| Liver Disease (%) | 5920 (64.9) | 2092 (68.8) | 1995 (65.6) | 1833 (60.3) | **< 0.001** |
| Myocardial Infarct (%) | 1271 (13.9) | 231 (7.6) | 418 (13.7) | 622 (20.4) | **< 0.001** |
| Congestive Heart Failure (%) | 3875 (42.5) | 1000 (32.9) | 1376 (45.2) | 1499 (49.3) | **< 0.001** |
| Cerebrovascular Disease (%) | 2034 (22.3) | 724 (23.8) | 688 (22.6) | 622 (20.4) | **0.006** |
| Chronic Pulmonary Disease (%) | 1001 (11.0) | 338 (11.1) | 356 (11.7) | 307 (10.1) | 0.125 |
| Malignant Cancer (%) | 1961 (21.5) | 690 (22.7) | 653 (21.5) | 618 (20.3) | 0.079 |
| Sepsis (%) | 6311 (69.2) | 1894 (62.3) | 2067 (68) | 2350 (77.3) | **< 0.001** |
| **Laboratory results** |  |  |  |  |  |
| WBC (K/uL) | 13.6 ± 11.9 | 13.1 ± 8.0 | 13.2 ± 10.7 | 14.3 ± 15.6 | **< 0.001** |
| RBC (K/uL) | 3.5 ± 0.8 | 3.6 ± 0.7 | 3.5 ± 0.8 | 3.3 ± 0.8 | **< 0.001** |
| Hemoglobin (g/dL） | 10.4 ± 2.2 | 10.7 ± 2.1 | 10.5 ± 2.3 | 10.0 ± 2.3 | **< 0.001** |
| Sodium (mEq/L) | 138.2 ± 5.7 | 138.0 ± 5.5 | 138.5 ± 5.3 | 138.2 ± 6.2 | **< 0.001** |
| Potassium (mEq/L) | 4.3 ± 0.8 | 4.1 ± 0.7 | 4.3 ± 0.7 | 4.5 ± 0.9 | **< 0.001** |
| Calciumtotal (mg/dL) | 8.3 ± 0.9 | 8.4 ± 0.8 | 8.3 ± 0.8 | 8.2 ± 0.9 | **< 0.001** |
| Chloride (mEq/L) | 103.7 ± 6.9 | 103.3 ± 6.6 | 104.5 ± 6.5 | 103.3 ± 7.6 | **< 0.001** |
| Glucose (mg/dL) | 154.1 ± 76.9 | 144.6 ± 60.5 | 156.2 ± 80.2 | 161.6 ± 86.7 | **< 0.001** |
| Total Bilirubin (mg/dL) | 1.4 ± 2.9 | 1.0 ± 1.8 | 1.2 ± 2.5 | 1.9 ± 3.9 | **< 0.001** |
| BUN (mg/dL) | 34.1 ± 25.5 | 21.5 ± 12.6 | 30.7 ± 18.9 | 50.1 ± 31.7 | **< 0.001** |
| **Interventions** |  |  |  |  |  |
| Ventilation (%) | 8089 (88.7) | 2663 (87.6) | 2728 (89.7) | 2698 (88.7) | **0.032** |
| CRRT (%) | 778 (8.5) | 41 (1.3) | 142 (4.7) | 595 (19.6) | **< 0.001** |
| Vasopressin (%) | 5767 (63.2) | 1650 (54.3) | 1959 (64.4) | 2158 (70.9) | **< 0.001** |
| Diuretic (%) | 2296 (25.2) | 700 (23) | 898 (29.5) | 698 (22.9) | **< 0.001** |
| ACEI (%) | 1820 (19.9) | 689 (22.7) | 664 (21.8) | 467 (15.4) | **< 0.001** |
| Statins (%) | 1807 (19.8) | 614 (20.2) | 658 (21.6) | 535 (17.6) | **< 0.001** |
| **Hospital stays** | 15.5 ± 14.5 | 14.9 ± 13.4 | 15.2 ± 13.3 | 16.3 ± 16.5 | **< 0.001** |
| **ICU stay** | 6.6 ± 7.5 | 6.2 ± 7.2 | 6.6 ± 7.6 | 6.9 ± 7.8 | **< 0.001** |
| **In-icu mortality (%)** | 2048 (22.4) | 439 (14.4) | 573 (18.8) | 1036 (34.1) | **< 0.001** |
| **In-hospital mortality (%)** | 732 (8.0) | 190 (6.2) | 226 (7.4) | 316 (10.4) | **< 0.001** |
| **28-day mortality (%)** | 1977 (21.7) | 432 (14.2) | 559 (18.4) | 986 (32.4) | **< 0.001** |

Data are presented as the mean ± SD or median (IQR) for skewed variables, and numbers (proportions) for categorical variables. EASIX values were multiplied by 10 for analysis. P values less than 0.05 are expressed in bold.
EASIX, Endothelial Activation and Stress Index; bpm: beats per minute; MBP: Mean Blood Pressure; SOFA: Sequential Organ Failure Assessment score; CCI: Charlson Comorbidity Index; APSIII: Acute Physiology and Chronic Health Evaluation III score; OASIS: Outcome and Severity of Illness Score; WBC: White Blood Cell count; RBC: Red Blood Cell count; SCR: Serum Creatinine; BUN: Blood Urea Nitrogen. ACEI: angiotension converting enzyme inhibitors.
