# Supplementary material for: The association between endothelial activation and stress Index and the development and prognosis of acute kidney injury in elderly patients with critical illness
Source: Ren Fail. 2025 Nov 4;47(1):2577174. doi: 10.1080/0886022X.2025.2577174 (PMC12587800; doi:10.1080/0886022X.2025.2577174)
Supplement: Manuscript_Figures_Tables_SupplFiles_KZou.zip.zip [file IRNF_A_2577174_SM7691.zip › figures, tables and supplementary files/Table 2.docx]

**Table 2. Multivariable Cox regression analysis of clinical outcomes.**

|  | Unadjusted | |  | Model 1 | |  | Model 2 | |
| --- | --- | --- | --- | --- | --- | --- | --- | --- |
| Variable | HR（95% CI） | *p* value |  | HR（95% CI） | *p* value |  | HR（95% CI） | *p* value |
| **Primary outcomes** |  |  |  |  |  |  |  |  |
| **28-day mortality** |  |  |  |  |  |  |  |  |
| T1 | 1(Ref) |  |  | 1(Ref) |  |  | 1(Ref) |  |
| T2 | 1.24 (1.09~1.40) | **0.001** |  | 1.25 (1.10~1.41) | **0.001** |  | 1.22 (1.07~1.40) | **0.003** |
| T3 | 2.12 (1.89~2.37) | **<0.001** |  | 2.22 (1.98~2.49) | **<0.001** |  | 1.69 (1.47~1.95) | **<0.001** |
| P for trend |  | **<0.001** |  |  | **<0.001** |  |  | **<0.001** |
| **Secondary outcomes** |  |  |  |  |  |  |  |  |
| **In-icu mortality** |  |  |  |  |  |  |  |  |
| T1 | 1(Ref) |  |  | 1(Ref) |  |  | 1(Ref) |  |
| T2 | 1.22 (1.08~1.38) | **0.002** |  | 1.23 (1.09~1.40) | **0.001** |  | 1.16 (1.02~1.32) | **0.029** |
| T3 | 2.03 (1.81~2.27) | **<0.001** |  | 2.11 (1.88~2.36) | **<0.001** |  | 1.55 (1.34~1.79) | **<0.001** |
| P for trend |  | **<0.001** |  |  | **<0.001** |  |  | **<0.001** |
| **In-hospital mortality** |  |  |  |  |  |  |  |  |
| T1 | 1(Ref) |  |  | 1(Ref) |  |  | 1(Ref) |  |
| T2 | 1.16 (0.96~1.41) | 0.123 |  | 1.17 (0.96~1.42) | 0.121 |  | 1.23 (0.99~1.51) | 0.051 |
| T3 | 1.48 (1.23~1.77) | **<0.001** |  | 1.58 (1.32~1.90) | **<0.001** |  | 1.42 (1.13~1.79) | **0.003** |
| P for trend |  | **<0.001** |  |  | **<0.001** |  |  | **<0.001** |
| **90-day mortality** |  |  |  |  |  |  |  |  |
| T1 |  |  |  |  |  |  |  |  |
| T2 | 1.05 (0.94~1.18) | 0.400 |  | 1.03 (0.92~1.16) | 0.627 |  | 1.04 (0.92~1.18) | 0.523 |
| T3 | 1.32 (1.19~1.47) | **<0.001** |  | 1.37 (1.23~1.52) | **<0.001** |  | 1.16 (1.02~1.32) | **0.022** |
| P for trend |  | **<0.001** |  |  | **<0.001** |  |  | **0.016** |
| **365-day mortality** |  |  |  |  |  |  |  |  |
| T1 |  |  |  |  |  |  |  |  |
| T2 | 1.05 (0.94~1.18) | 0.367 |  | 1.03 (0.92~1.15) | 0.578 |  | 1.04 (0.93~1.17) | 0.482 |
| T3 | 1.32 (1.2~1.46) | **<0.001** |  | 1.36 (1.23~1.51) | **<0.001** |  | 1.16 (1.02~1.31) | **0.020** |
| P for trend |  | **<0.001** |  |  | **<0.001** |  |  | **0.015** |

Hazard ratios (HR) and 95% confidence intervals (CI) are presented for 28-day mortality, in-ICU mortality, in-hospital mortality, 90-day mortality, and 365-day mortality.

**Unadjusted**: Crude model without covariate adjustment.

**Model 1**: Adjusted for age, sex, and ethnicity.

**Model 2**: Adjusted for age, sex, ethnicity, weight, vital signs (heart rate, respiration rate, SpO₂, mean blood pressure), scoring systems (SOFA, CCI, APSIII, OASIS), comorbidities (hypertension, diabetes, liver disease, myocardial infarct, congestive heart failure, cerebrovascular disease, chronic pulmonary disease, malignant cancer, sepsis), laboratory results (WBC, RBC, hemoglobin, sodium, potassium, calcium, chloride, glucose, total bilirubin, BUN), and interventions (ventilation, CRRT, vasopressin, diuretic, ACEI, Statins).
P-values less than 0.05 are expressed in bold.
