# Supplementary material for: The association between endothelial activation and stress Index and the development and prognosis of acute kidney injury in elderly patients with critical illness
Source: Ren Fail. 2025 Nov 4;47(1):2577174. doi: 10.1080/0886022X.2025.2577174 (PMC12587800; doi:10.1080/0886022X.2025.2577174)
Supplement: Manuscript_Figures_Tables_SupplFiles_KZou.zip.zip [file IRNF_A_2577174_SM7691.zip › figures, tables and supplementary files/Table 3.docx]

**Table 3 Threshold effect analysis.**

| Item | Adjusted HR (95%CI) | P value |
| --- | --- | --- |
| Fitting by the two-piecewise Cox proportional risk model inflection point | 174.855 (167.759~181.952) |  |
| EASIX＜174.855 | 1.0034 (1.002~1.0049) | **< 0.001** |
| EASIX≥174.855 | 1 (0.9992~1.0008) | 0.9709 |
| Likelihood Ratio test |  | **<0.001** |

Note: The data were adjusted for all potential confounding variables.
